# Molecular understanding of cation effects on double layers and their significance to CO-CO dimerization

**DOI:** 10.1093/nsr/nwad105

**Published:** 2023-04-20

**Authors:** Jia-Bo Le, Ao Chen, Yongbo Kuang, Jun Cheng

**Affiliations:** Key Laboratory of Advanced Fuel Cells and Electrolyzers Technology of Zhejiang Province, Ningbo Institute of Materials Technology and Engineering, Chinese Academy of Sciences, Ningbo 315201, China; State Key Laboratory of Physical Chemistry of Solid Surfaces, iChEM, College of Chemistry and Chemical Engineering, Xiamen University, Xiamen 361005, China; Key Laboratory of Advanced Fuel Cells and Electrolyzers Technology of Zhejiang Province, Ningbo Institute of Materials Technology and Engineering, Chinese Academy of Sciences, Ningbo 315201, China; State Key Laboratory of Physical Chemistry of Solid Surfaces, iChEM, College of Chemistry and Chemical Engineering, Xiamen University, Xiamen 361005, China; Innovation Laboratory for Sciences and Technologies of Energy Materials of Fujian Province (IKKEM), Xiamen 361100, China

**Keywords:** cation effect, electrocatalysis, electric double layer, CO-CO dimerization, *ab initio* molecular dynamics

## Abstract

Cation effects have been shown in numerous experiments to play a significant role in electrocatalysis. To understand these effects at the molecular level, we systematically investigate the structures and capacitances of electric double layers with a variety of cations as counter charges at Pt(111)-CO_ad_/water interfaces with *ab initio* molecular dynamics. It is encouraging to find that the computed Helmholtz capacitances for different cations are in quantitative agreement with experiments, and that the trend of cation effects on capacitances shows clear correlation with the structures of interface cations of differing sizes and hydration energies. More importantly, we demonstrate the Helmholtz capacitance as the key descriptor for measuring the activity of CO-CO dimerization, the rate-determining step for C_2+_ formation in electroreduction of CO and CO_2_. Our work provides atomistic insights into cation effects on electric double layers and electrocatalysis that are crucial for optimizing electrode and electrolyte materials.

## Teaser

Unraveling the microscopic mechanism of the cation effect on the selectivity of C_2_ product in CO reduction reaction using ab initio molecular dynamics.

## INTRODUCTION

Electrocatalytic reactions occurring at solid-liquid interfaces enable the reversible conversion of chemical energy and electricity [1,2]. To improve the efficiency of electrocatalysis, enormous efforts have been devoted to developing new catalysts and optimizing their morphologies. Recently, it has been shown that not only electrode materials and solvents, but also cations in electrolyte solutions have profound effects on the activity and selectivity of a large variety of electrocatalytic reactions, such as the hydrogen evolution reaction [3], hydrogen oxidation reaction (HOR) [4], oxygen reduction reaction (ORR) [4,5], CO oxidation [6], CO reduction reaction (CORR) [7–9] and CO_2_ reduction reaction (CO_2_RR) [10–13]. Consequently, many studies have focused on understanding the mechanisms of the cation effects on electrocatalytic reactions.

Generally speaking, cations at electric double layers (EDLs) can influence the strengths of interfacial electric fields, the local environments of active sites, the structures of interface water, local pH values, etc., based on which various mechanisms have been proposed to account for the cation effects on different electrocatalytic reactions [14]. For instance, Markovic and co-workers [4] found that the trend of the cation-dependent activity of ORR, HOR and methanol oxidation on the Pt electrode follows the order Cs^+^ > Rb^+^ > K^+^ > Na^+^ >> Li^+^, which is opposite to the order of the interaction energies between cations and surface-adsorbed OH, suggesting that the cations at interfaces block the active surface sites. Waegele and co-workers [8] reported that, for CORR on the Cu electrode, increasing the size of alkylammonium ions largely decreases the ethylene selectivity in favor of by-product H_2_, and proposed that the large size of alkylammonium ions decreases the hydrogen bonding between surface CO and water, thus hindering CO dimerization. More recently, Koper and co-workers [10] revealed that CO_2_RR cannot proceed on Ag, Cu and Au electrodes unless using metal ions as supporting electrolytes, and suggested that the short-range electrostatic interaction with partially desolvated cations is key to the stabilization of the reaction intermediate CO$_2^-$. Usually, there are a few possible explanations proposed for the cation effects of electrocatalytic reactions such as CORR [7,8,15] and CO_2_RR [10–12], but the underlying mechanism is still in debate due to a lack of molecular-level understanding of cation effects on EDLs. In order to reveal the molecular origin of cation effects, several fundamental questions need to be addressed; for example, how the cations alter the structures of interface water and potential distribution, and how they interact with reaction intermediates and affect surface reactions.

Complementary to experimental techniques [5,14,16–18], *ab initio* modeling of EDLs is an effective approach to investigate the cation effects at the molecular level. In particular, *ab initio* molecular dynamics (AIMD) has been demonstrated in recent years to be a useful tool to explore the microscopic structures of EDLs. For example, it was revealed by AIMD simulations that the orientations of interface water molecules are strongly dependent on the electrode potential [19–23]; the OH bonds of water change the direction from the electrode surface to bulk solution when shifting the potential to positive. Moreover, it was shown that the interaction mode between cations and surface adsorbates is also potential dependent [24,25]. Cations, especially with large size, tend to undergo partial desolvation when increasing the strength of the electric field at interfaces, and as a result, the interaction between cations and surface adsorbates changes from the long-range electrostatic attraction to short-range coordination. Recently, Yang and co-workers [26] showed with AIMD that the nature of cations is also important to the interaction with surface adsorbates; cations with lower hydration energies (i.e. Cs^+^) are easier to desolvate and coordinate with surface adsorbates.

To elucidate the cation effects on EDL structures and capacitances, and their importance to electrocatalysis, we employ AIMD to systematically simulate a series of electrified Pt(111)-CO_ad_/water interfaces with a variety of alkali metal and alkylammonium ions. The Pt(111)-CO_ad_/water interface is chosen as the model system for this study because of the following considerations, although Pt is not a promising catalyst for CORR and CO_2_RR. First, the double-layer capacitances of Pt-CO_ad_/water interfaces with different cations have been measured in a recent experimental work [25], thus enabling direct comparison between experiment and theoretical computation. Second, the coverage and adsorption pattern of CO on the Pt(111) surface have been well characterized by electrochemical scanning tunneling microscopy (EC-STM) [27], which helps build realistic interface models. Third, since we consider high coverage of surface CO in this study and the metal surface is not in direct contact with the electrolyte, the Pt-CO_ad_ surface should not differ much from the Cu-CO_ad_ surface.

Based on extensive AIMD simulations, we propose that at the same potential conditions the EDL may be altered in various ways by cations with different sizes and hydration energies, such as varying surface charge densities, hydrogen bonding between surface CO and interface water molecules, and coordination between surface CO molecules and cations. We also show that the surface charge densities and coordination with water and cations can have significant impact on CO dimerization, a key step in CORR and CO_2_RR. What is interesting is that we demonstrate that all these structural factors can be wrapped into the sole descriptor of Helmholtz capacitance for measuring the cation effects on the activity of CO dimerization. We believe that this finding is of great importance for understanding the microscopic mechanisms of cation effects on EDLs, and developing strategies for improving electrocatalysis.

## RESULTS

### Helmholtz capacitances

**Figure 1.**
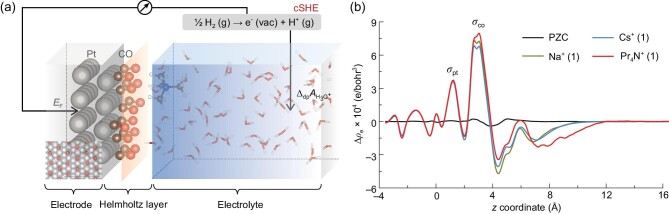
(a) Model of an electrified Pt(111)-CO_ad_/water interface. The surface has a Pt(111)(2×2)-3CO adlayer structure with a CO coverage of 3/4 MLs. The Helmholtz plane of the electric double layer passes through the centers of the counter ions. Pt, C, N, O and H atoms in the model are colored gray, brown, blue, red and white, respectively. The electrode potential of the interface model is determined by the computational standard hydrogen electrode (cSHE) method. Here *E*_F_ and $\Delta _\mathrm{dp}A_\mathrm{H_3O^+}$ denote the computed Fermi level of the interface model and the reference deprotonation energy of a hydronium ion in the bulk water, respectively. (b) Distributions of the planar average of excess charges (Δρ_e_) along the *z* coordinate. Here Δρ_e_ is calculated with $\Delta \rho _\mathrm{e} = \rho _\mathrm{interface}-\rho _\mathrm{Pt(111)-CO_{ad}}-\rho _\mathrm{water}-\rho _\mathrm{ion}$, where ρ_interface_, $\rho _\mathrm{Pt(111)-CO_{ad}}$, ρ_water_ and ρ_ion_ represent the electronic densities of the interface, neutral Pt(111)-CO_ad_ surface, water and ions at valence states zero, respectively. Excess charges on the surface are separated into two sub-layers, on the Pt surface (σ_Pt_) and on the oxygen side of the CO adlayer (σ_CO_).

Figure 1(a) shows the model setup of an electrified Pt(111)-CO_ad_/water interface. Based on EC-STM measurements [27], the surface coverage of CO in the interface models is set to 3/4 monolayers (MLs), and they are arranged with a (2 × 2)-3CO pattern. Note that in the interface models, the electronic charges on the Pt(111)-CO_ad_ surfaces are fully compensated by the counter ions kept in the vicinity of the surfaces, resulting in compact Helmholtz layers. The diffuse layer of the EDL is absent in this setup, and thus our models correspond to high ionic strength conditions when the diffuse layers are suppressed [28,29]. EDLs with alkali metal ions and alkylammonium ions, including Li^+^, Na^+^, Rb^+^, Cs^+^, Me_4_N^+^, Et_4_N^+^ and Pr_4_N^+^, are modeled at the same surface charge density $\sigma = -14.6\,\mu$C/cm^2^, as shown in Fig. S1 within the online supplementary material. To study the effect of the interfacial ionic concentration on double-layer capacitance, we have also modeled interfaces with extra Cs^+^-Cl^−^ ion pairs. All interface models are simulated with AIMD for dozens of picoseconds to sufficiently sample the dynamic structures of the electrified interfaces, and the cation effects on the EDL structures and capacitances are then investigated. It should be mentioned that the surface pattern of the CO adlayer is well kept during the AIMD simulations (see Fig. S6 within the online supplementary material), indicating that the structures of the electrode surfaces are stable and the same for all interface models. More details about the EDL models and computational setup can be found in the Methods section and Section S1 of the online supplementary material.

The electrode potentials of all interface models are calculated using the recently developed computational standard hydrogen electrode (cSHE) method [30–33]. As illustrated in Fig. 1(a), the computed Fermi levels of metal electrodes are referenced to the solvation free energy of a proton (i.e. deprotonation energy of a solvated hydronium), which can be converted into the SHE scale for direct comparison with experiment. A brief description of the cSHE method is summarized in Section S2 of the online supplementary material. As listed in Table 1, the computed potential of zero charge (PZC) of the Pt(111)-CO_ad_/water interface is 1.08 ± 0.06 V vs SHE, which is almost the same as the experimental data [34,35]. The electrode potentials of all the EDL models are also computed. It was reported that the capacitance of the Pt-CO_ad_/water interface is independent of the electrode potential [25,35]. Thus, we can estimate the Helmholtz capacitance (*C*_H_) of each EDL model by taking *C*_H_ = Δσ/Δ*U* with PZC as a reference. It is encouraging to find from Table 1 that nearly all computed *C*_H_ are in quantitative agreement with experiment [25]. This is a strong indication that our calculation is a good representation of electrified Pt-CO_ad_/water interfaces. We note that the computed *C*_H_ of alkali metal ions appear less accurate than that of alkylammonium ions, particularly for those with large sizes, e.g. Rb^+^ and Cs^+^. In a very recent work, Waegele and co-workers [36] proposed that cations (e.g. Cs^+^) with lower hydration energies are more concentrated at the interface than those with higher hydration energies (e.g. Li^+^). To understand the effect of interface cation concentration, we model an interface with two Cs^+^ and one Cl^−^ ions. It is shown in Table 1 that the electrode potential of model Cs^+^(2) + Cl^−^(1) is 0.12 V more positive than model Cs^+^(1), and the computed *C*_H_ increases from 11.7 to 13.0 $\mu$F/cm^2^, becoming closer to the experimental value (14.3 $\mu$F/cm^2^). Thus, our results indeed support the finding reported by Waegele and co-workers [36]. Overall, comparing the tabulated values of *C*_H_ and ion radii, several key features can be obtained: (i) the EDL capacitances for alkylammonium ions are noticeably smaller than those for alkali metal ions; (ii) for alkylammonium ions, *C*_H_ decreases with increasing ion size; (iii) the trend for alkali metal ions is somewhat less clear, but the experimental *C*_H_ shows a slight increasing tendency with increasing ion size.

**Table 1.** Calculated electrode potentials (*U*) and Helmholtz capacitances (*C*_H_) of Pt(111)-CO_ad_/water interfaces with different ions.

	*r*_ion_	σ	*U* vs SHE	*C*_H_	*C*_ad_	*C*_sol_
Model	(Å)	($\mu$C/cm^2^)	(V)	($\mu$F/cm^2^)	($\mu$F/cm^2^)	($\mu$F/cm^2^)
PZC	–	0	1.08 ± 0.06 (1.10)	–	–	–
Li^+^(1)	0.69	−14.6	−0.30 ± 0.07	10.6 (10.6)	44.5	13.9
Na^+^(1)	1.02	−14.6	−0.02 ± 0.07	13.3 (12.0)	44.5	19.0
Rb^+^(1)	1.49	−14.6	−0.13 ± 0.06	12.1 (14.5)	44.5	16.6
Cs^+^(1)	1.70	−14.6	−0.17 ± 0.05	11.7 (14.3)	44.5	15.9
Cs^+^(2) + Cl^−^(1)	–	−14.6	−0.05 ± 0.06	13.0 (14.3)	44.5	18.4
Me_4_N^+^(1)	2.79	−14.6	−0.37 ± 0.06	10.1 (10.4)	44.5	13.1
Et_4_N^+^(1)	3.36	−14.6	−0.54 ± 0.05	9.0 (8.6)	44.5	11.3
Pr_4_N^+^(1)	3.78	−14.6	−1.03 ± 0.06	6.9 (6.0)	44.5	8.2

Data in the parentheses are the experimentally measured PZC of the Pt(111)-CO_ad_/water interface [34,35] and double-layer capacitances of the (poly)Pt-CO/water interfaces [25]. Here *C*_H_ is decomposed into two components, the CO-adlayer-induced capacitance (*C*_ad_) and water-dielectric-screening-induced capacitance (*C*_sol_), which are connected in series. The *r*_ion_ is determined from crystal radii for alkali metal ions [37] and van der Waal’s volume for alkylammonium ions [38].

### Residence locations and local structures of cations at interfaces

It can be seen from Fig. 1(b) that the CO molecules on the Pt(111) surface act as a dielectric layer with a finite dielectric constant. Surface charge is separated into two components, charges on Pt (σ_Pt_) and charges on CO (σ_CO_). Similar results have been reported by Sundararaman and co-workers [39]. As a consequence, in addition to the capacitance from water dielectric screening (*C*_sol_), the *C*_H_ of the Pt(111)-CO_ad_/water interface is composed of another component, the CO-adlayer-induced capacitance (*C*_ad_). According to the mathematical formulation derived in Section S3 of the online supplementary material, we can show that *C*_ad_ and *C*_sol_ are connected in series, and that *C*_ad_ is only dependent on the partition of surface charge on the Pt(111) surface. Figure 1(b) and Fig. S3(c) within the online supplementary material indicate that the nature of cations does not affect the partition of σ_Pt_. This means that *C*_ad_ is a constant for all our modeled interfaces, 44.5 $\mu$F/cm^2^, and the corresponding *C*_sol_ values are then calculated as listed in Table 1. In addition, we find that the surface coverage of CO (θ_CO_) significantly affects the magnitude of σ_Pt_ (see Fig. S14 within the online supplementary material), and thus θ_CO_ is also an important property for the *C*_H_ of the Pt(111)-CO_ad_/water interface that should not be ignored.

**Figure 2.**
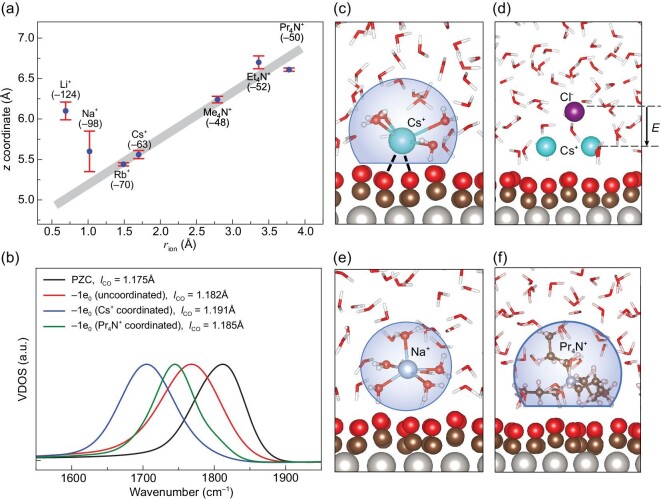
(a) Residence locations of counter ions (*z* coordinate) at the electrified Pt(111)-CO_ad_/water interfaces as a function of their corresponding ionic radii (*r*_ion_). The hydration energies of the ions (in Kcal/mol) are given in parentheses [4,42]. The linearly correlated data points are shaded with gray blocks. The standard error bars are shown for the *z* coordinates of cations. (b) Comparison of vibrational density of states (VDOS) of hollow-site CO on the Pt(111) surface at four different electrochemical environments: PZC condition, as well as negatively charged surfaces with CO not coordinating with ion or water, CO coordinating with Cs^+^ and CO coordinating with Pr_4_N^+^. The corresponding bond lengths of CO (*l*_CO_) at different conditions are calculated. VDOS spectra are calculated with the Fourier transformation of the velocity-velocity autocorrelation function from AIMD trajectories. (c–f) Representative snapshots of local structures of models Cs^+^(1), Cs^+^(2) + Cl^−^(1), Na^+^ and Pr_4_N^+^(1) from AIMD simulations. Pt, C, O, H, Na, Cs, Cl and N atoms are colored gray, brown, red, white, light steel blue, cyan, purple and blue, respectively.

It is well established that the solvent capacitance layer resembles a parallel-plate capacitor [23,28,40], and its capacitance can be expressed as *C*_sol_ = ϵ_sol_ϵ_0_/*l*_sol_, where *l*_sol_ and ϵ_sol_ represent the width and dielectric constant of the solvent capacitance layer, respectively, and ϵ_0_ is the dielectric constant of vacuum. Since *C*_sol_ is inversely proportional to *l*_sol_, the variation in *C*_sol_ for different cations is often attributed to the difference in the widths of the solvent capacitance layer [25,40,41]. To verify this hypothesis, we analyze the residence locations (i.e. *z* coordinate with reference to the Pt(111) surface) of the cations at the electrified Pt(111)-CO_ad_/water interfaces. Figure 2(a) plots the *z* coordinates of cations as a function of their radii (*r*_ion_). It is interesting to find that, for cations with relatively low hydration energies (below 70 Kcal/mol; see the numbers in parentheses in Fig. 2(a)), such as Rb^+^ and Me_4_N^+^, their *z* coordinates generally become larger with increasing *r*_ion_, and shows a good linear correlation. On the other hand, cations with high hydration energies (∼100 Kcal/mol or above), i.e. Li^+^ and Na^+^, are apparently off and above the trend line.

To understand the linear correlation between the *z* coordinate and *r*_ion_ for cations with low hydration energies and the abnormality for alkali metal ions with high hydration energies, we investigate the local structures of these cations at the electrified interfaces. Typical snapshots taken from AIMD trajectories are shown in Fig. 2(c–f). We first note that, for ions like Cs^+^ and Pr_4_N^+^ having low hydration energies, their solvation shells are partially removed, with ions directly in contact with the CO adlayer. It can thus be understood why the *z* coordinates of these ions increase along with increasing ion size. It should be mentioned that the *r*_ion_ values of alkylammonium ions are estimated from the van der Waal’s volume by assuming that they are spheres [38]. The Pr_4_N^+^ ions tend to orient three side chains towards the surface, as seen in Fig. 2(f). This means that the distance of the N atoms of Pr_4_N^+^ from the surface will be shorter than the estimated *r*_ion_, and as a consequence, in Fig. 2(a) the data point of Pr_4_N^+^ is slightly below the trend line. In addition, different from the so-called ‘cation-specific adsorption’ [36], we find that the interaction between these cations and CO is purely electrostatic in nature. No partial charge transfer occurs between cations and the metal surface, which is evidenced by the electron difference plot shown in Fig. 1(b), electronic density of states (DOS) shown in Fig. S4 within the online supplementary material and Mulliken charges of cations listed in Table S1 within the online supplementary material. For cations with higher hydration energies like Na^+^, our AIMD calculations show that their solvation shells are complete at the electrified interfaces. As shown in Fig. 2(e), those cations do not directly contact with surface CO, and thereby they stay further away from the surface than estimated from the linear correlation (the shaded area in Fig. 2(a)).

We then compare the polarization effects of CO on the Pt(111) surface. As shown in Fig. 2(b), the vibrational frequency of the hollow-site CO shows a red shift in the presence of an interfacial electric field due to the well-known Stark effect, and the C–O bond length (*l*_CO_) is accordingly increased. Moreover, upon coordination with metal cations such as Cs^+^, the CO frequency undergoes a further red shift owing to the short-range electrostatic interaction, and similar polarization effects have also been observed in recent work [43,44]. In comparison, the short-range electrostatic interaction with CO of Cs^+^ is much stronger than that of Pr_4_N^+^, and it is because the excess charges on Cs^+^ are more localized than Pr_4_N^+^ (see Fig. S5(h) within the online supplementary material). The ion distributions at the interface in the presence and absence of extra ion pairs are also compared. As shown in Fig. 2(c–d) and Fig. S9 within the online supplementary material, the distributions of Cs^+^ in two models are almost the same, and due to the electrostatic repulsion, Cl^−^ stays further away from the surface compared to Cs^+^. As a consequence, the extra Cs^+^-Cl^−^ pair forms a net dipole at the interface, which is expected to increase the electrode potential and *C*_H_, in line with AIMD results.

### Structures of interface water

**Figure 3.**
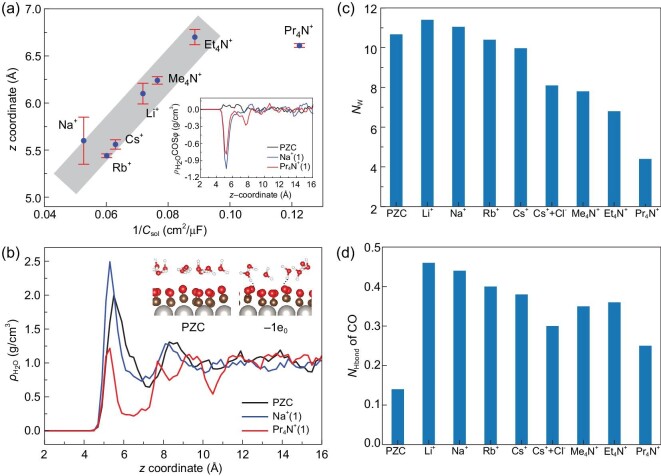
(a) The *z* coordinates of cations as a function of their corresponding 1/*C*_sol_. The linearly correlated data points are shaded with gray blocks. The standard error bars are shown for the *z* coordinates of cations. The inset shows the distributions of the dipole orientation of interface water molecules ($\rho _\mathrm{H_{2}O}$cosϕ) along the surface normal. Here ϕ is defined as the angle between the bisector of water and the surface normal. (b) The distribution profiles of water density ($\rho _\mathrm{H_{2}O}$) along the surface normal (*z* coordinate) at electrified interfaces with different cations. The insets show two typical snapshots of the Pt(111)-CO_ad_/water interface when the surface is neutral and charged. (c) Count of interface water molecule count (*N*_w_) at electrified interfaces with different ions. (d) Averaged number of hydrogen bonds (*N*_Hbond_) between surface CO and water.

By relating the residence locations of cations to their corresponding solvent layer capacitances (*C*_sol_), we find from Fig. 3(a) that, for the majority of cations, the *z*-coordinate linearly correlates with the inverse of *C*_sol_, which is indeed consistent with the expectation of treating the solvent capacitance layer as a parallel-plate capacitor. This is remarkable because this correlation is valid for ions having both full and partial solvation shells. A minor exception is the bulky Pr_4_N^+^ ion; considering its large size, one would expect that a significant amount of water may be excluded by the ions at the interface, leading to a different dielectric constant of the interface (see below).

We then investigate the cation effects on the structures of interface water at Pt(111)-CO_ad_/water interfaces. It can be seen from Fig. 3(b) (see also Fig. S10 within the online supplementary material for the results of other cations) that the density distributions of water ($\rho _\mathrm{H_{2}O}$) are rather different with different cations as counter ions at the electrified interfaces. The number of interface water molecules (*N*_w_) is counted and averaged along the AIMD trajectories of the interface models and presented in Fig. 3(c). It is interesting to note that introducing alkali metal ions at the interfaces hardly changes the number of interface water molecules *N*_w_. In contrast, *N*_w_ decreases significantly by introducing alkylammonium ions at the interfaces. In particular, for Pr_4_N^+^, *N*_w_ reduces to less than half of those in models with alkali metal ions. Fewer interface water molecules implies that the dielectric constant ϵ_sol_ of the interface for Pr_4_N^+^ would be considerably smaller than those with alkali metal ions, consistent with the low *C*_sol_ for Pr_4_N^+^ measured in experiment [25].

The inset of Fig. 3(a) shows the distributions of the dipole orientation of interface water molecules ($\rho _\mathrm{H_{2}O}$cosϕ) at PZC and negative potentials with different cations (see Fig. S11 within the online supplementary material for the results of other cations). At negatively charged surfaces, the interface water molecules tend to orient with their O–H bonds pointing towards the surface (‘H-down’ water), owing to electrostatic attraction between the partially positively charged H atoms and the surfaces [45]. Similar configurations of the interface water molecules have been observed at other electrified metal interfaces [19,20,23,24]. As shown in the insets of Fig. 3(b), there are also more hydrogen bonds formed between interface water and surface CO molecules at negatively charged interfaces compared to the PZC condition. It can be seen from Fig. 1(b) that most of the negative surface charge is located at the O atoms of the surface CO, strengthening the hydrogen bonding between the surface CO and ‘H-down’ water.

The averaged number of hydrogen bonds (*N*_Hbond_) accepted by each CO is illustrated in Fig. 3(d) for different interface models. It is known that the neutral CO is hydrophobic, and thus there are almost no hydrogen bonds formed between CO and water at PZC. After negatively charging the surface, we find that *N*_Hbond_ increases considerably for all the interfaces with different counter ions, indicating that *N*_Hbond_ is dependent of the surface charge density. Figure 3(d) also shows that the cation size has a significant impact on *N*_Hbond_. In the EDLs with small alkali metal ions like Li^+^ and Na^+^, each surface CO can form on average 0.4–0.5 hydrogen bonds with water. Also, it can be shown that *N*_Hbond_ decreases when the ion size increases, and in particular for bulky Pr_4_N^+^, *N*_Hbond_ reduces to only 0.25. This can be readily understood: the large size of Pr_4_N^+^ excludes water from the interface, thus hindering direct contact between surface CO and water.

### Significance to electrocatalytic CO dimerization

We now explore how the cation-dependent Helmholtz capacitance and interface structure correlate with the activities of electrocatalytic reactions. The reaction examined here is the CO dimerization process, which is widely accepted as the rate-limiting step for the generation of C_2+_ products in CO_2_RR and CORR [7–9,26,46,47], and it has been known for several decades [7–9,11,15,41] that the nature of cations has prominent influence on the selectivity of C_2+_ products. It should be noted that Cu, rather than Pt, is a good catalyst for the CO dimerization reaction. However, at the electrochemical conditions that the metal surfaces are adsorbed with saturated CO, the properties of the Cu-CO_ad_/water interface would be very similar to the Pt-CO_ad_/water interface. Moreover, Fig. S12 within the online supplementary material supports the fact that the excess charge distribution on the Cu-CO_ad_ surface is almost the same as that on the Pt-CO_ad_ surface. Therefore, in this work we use the calculated structure and dielectric property of the Pt-CO_ad_/water interface to provide a qualitative understanding of the cation effects on the CO dimerization reaction that occurs at the Cu-CO_ad_/water interface.

**Figure 4.**
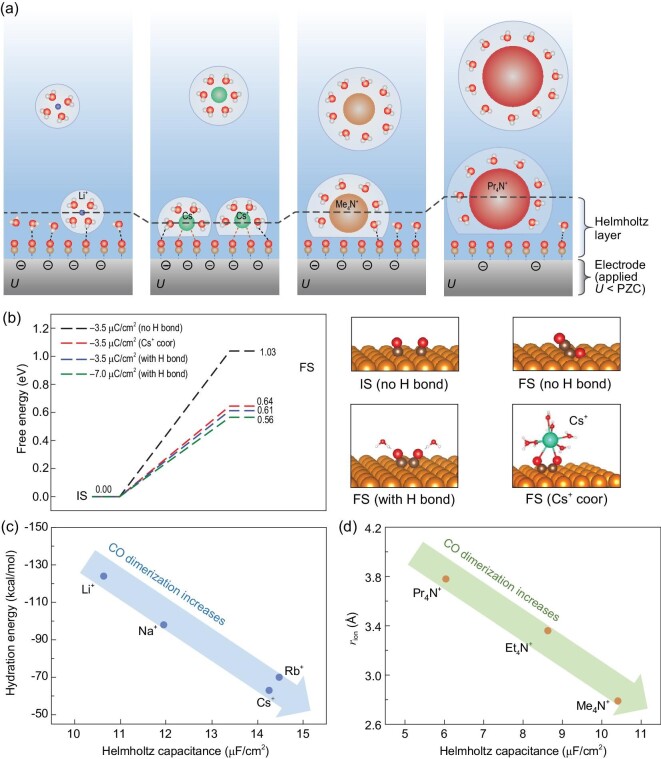
(a) Comparison of the cation-dependent Pt(111)-CO_ad_/water interfaces at the same applied electrode potential *U* (*U* < PZC). Different (negative) surface charge densities are shown with different negative sign amounts on the surfaces. (b) Calculated energies of the CO dimerization reaction on the Cu(100) surface under various conditions, i.e. different surface charge densities, and with or without coordination of water and the Cs^+^ ion. Configurations of OCCO* on the Cu(100) surface at different conditions are displayed. (c) Correlation between Helmholtz capacitances and hydration energies of alkali cations as a descriptor for measuring the selectivity of C_2+_ products in CORR [7]. (d) Correlation between Helmholtz capacitances and radii (*r*_ion_) of alkylammonium cations as a descriptor for measuring the selectivity of C_2+_ products in CORR [8].

In experiment the cation effects on the performance of electrocatalytic reactions are compared under the same electrode potential, while in our calculation above the cation effects on the structures of Pt(111)-CO_ad_/water interfaces are studied at the same surface charge density. Therefore, in order to better connect our calculation with the experimental results, we map our simulation results of the Pt(111)-CO_ad_/water interfaces at the same surface charge density (σ) to the same electrode potential (*U*). As illustrated in Fig. 4(a), at the same applied *U* (*U* < PZC), the amount of negative surface charge follows the order of magnitude of Helmholtz capacitance (*C*_H_; see Table 1), i.e. $\sigma _{\mathrm{Cs^+}}\sim \sigma _{\mathrm{Rb^+}}>\sigma _{\mathrm{Na^+}}> \sigma _{\mathrm{Li^+}}$ and $\sigma _{\mathrm{Me_4N^+}}>\sigma _{\mathrm{Et_4N^+}}> \sigma _{\mathrm{Pr_4N^+}}$.

An important structural consequence is that the negatively charged surface CO will attract hydrogen bonding from interface water, as shown in Fig. 3(d). Figure 4(b) shows that hydrogen bonding with water has significant impact on CO dimerization. In the absence of water hydrogen bonding, the CO dimerization energy on Cu(100) is 1.03 eV in a simplified charged surface model, and in contrast, it is significantly reduced to 0.61 eV with hydrogen bonding. A further increase in the negative surface charge can reduce the CO dimerization energy even more. Similar results were also observed by Norskov and co-workers [47]. Thus, we can propose the Helmholtz capacitance as a key descriptor for measuring the cation effects on the selectivity of C_2+_ products, following the order Cs^+^ > Rb^+^ > Na^+^ > Li^+^ in CO_2_RR and CORR and Me_4_N^+^ > Et_4_N^+^ > Pr_4_N^+^ in CORR, which agrees well with experiment [7,8,11]. The argument is as follows. The cation with a higher Helmholtz capacitance can result in higher negative surface charge density under the same electrode potential, as well as more hydrogen bonding between surface CO and water. Both contribute to the reduction of CO dimerization energy, facilitating the formation of C_2+_ products.

Furthermore, we can connect the microscopic structures of cations with their effects on CO dimerization. For alkali metal ions, the Helmholtz capacitance correlates less with the ion size, but strongly with the ion hydration energy, as shown in Fig. 4(c). Cs^+^ and Rb^+^ with low hydration energies can partially desolvate and get closer to the surface (see Fig. 4(a)), leading to a higher Helmholtz capacitance and thus easier CO dimerization. Besides, direct coordination of the ion with surface CO has an additional important effect of decreasing the CO dimerization energy (see Fig. 4(b) for Cs^+^). On the other hand, for the alkylammonium ions having similar solvation structures at the interface, it is expected that the Helmholtz capacitance inversely relates to the ionic radius, as shown in Fig. 4(d). Thus, a smaller alkylammonium ion will have a higher capacitance and is hence more favorable for CO dimerization. For larger alkylammonium ions, there is an extra negative effect that the hydrophobic alkyl groups exclude water from the interface, significantly reducing the hydrogen bonding with surface CO that is helpful to CO dimerization.

## CONCLUSIONS

To conclude, extensive AIMD simulations are performed to investigate the effects of a variety of counter cations differing in sizes and hydration energies, on the microscopic structures and capacitances of Pt(111)-CO_ad_/water interfaces. Our AIMD calculation is validated by comparing the computed Helmholtz capacitances with experiments; quantitative agreement is reached, and the general trend of cation effects can be reproduced. Furthermore, it is found that at the same electrode potentials the EDL with various cations as counter charges can differ in surface charge density, hydrogen bonding between surface CO and interface water, and coordination between surface CO and cations. Our calculation also shows that the surface charge density and coordination with water and cations can have important effects on the CO dimerization energy, which is a key factor determining the selectivity of C_2+_ products in CORR and CO_2_RR. Interestingly, we can use Helmholtz capacitance as the main descriptor to account for EDL differences caused by counter cations, and thus measure the activity of CO dimerization. We believe that our finding offers deeper understanding of cation effects on structures and reactivities of electrified interfaces, which is useful for improving the performance of electrocatalysts.

## METHODS

The Pt(111) surface was modeled by a *p*(4 × 4) periodic slab with four atomic layers. The surface coverage of adsorbed CO on Pt(111) was set to 3/4 ML, with 1/4 MLs on top sites and 1/2 MLs on hollow sites, forming a (2 × 2)-3CO adlayer. Both of the two Pt(111) surfaces were adsorbed with CO, and thus there is no net dipole. Enough vacuum region was modeled on top of the Pt(111) surface, and the overall size of the surface model was 11.246 × 11.246 × 40 Å^3^. A neutral Pt(111)-CO_ad_/water interface was modeled by fully filling the vacuum region in the Pt(111)-CO_ad_ surface model with water. The density of water in the bulk region was controlled to be ∼1 g/cm^3^. EDLs were modeled by introducing cations in the vicinity of the Pt(111)-CO_ad_ surface. Seven kinds of cations (Li^+^, Na^+^, Rb^+^, Cs^+^, Me_4_N^+^, Et_4_N^+^ and Pr_4_N^+^) were employed for modeling EDLs at the same surface charge density, σ = −14.6 $\mu$C/cm^2^. Moreover, to study the effect of interfacial ionic concentration on the double-layer capacitance, two extra Cs^+^-Cl^−^ ion pairs were added to the interface model Cs^+^(1). It should be mentioned that the Gouy-Chapman layers were not included in the modeled interfaces, and thus these EDL models correspond to the high concentration limits, where the surface charges are effectively screened within the Helmholtz layers.

AIMD simulations for Pt(111)-CO_ad_/water interfaces were performed by freely available software CP2K. Goedecker-Teter-Hutter pseudopotentials were used. The Gaussian basis set was double-ζ with one set of polarisation functions [48], and the energy cutoff was set to 400 Ry. The Becke-Lee-Yang-Parr (BLYP) functional was chosen [49] to describe the exchange-correlation effects in this work. The dispersion energy was corrected with Grimmer’s D3 method. The second generation Car-Parrinello molecular dynamics [50] method was used to sample the structures of interface models. The correction step was obtained by five iterations of the orbital transformation optimization, and the integration time for each step was 0.5 fs. The target temperature was set to 330 K. The Langevin friction coefficient (γ_*L*_) was set to 0.001 fs^−1^, and the intrinsic friction coefficients (γ_*D*_) were 2.2 × 10^−4^ fs^−1^ for H_2_O, CO and ions. The Pt slab was fixed during all the MD simulations. Because of the large size of the cells, only the Γ point in the reciprocal space was used in AIMD simulations. For each AIMD simulation, at least 5 ps (10 000 steps) of the trajectory was used to equilibrate the system, and then followed by a production period of 10–40 ps.

## Supplementary Material

nwad105_Supplemental_File
