# Supplementary material for: Molecular understanding of cation effects on double layers and their significance to CO-CO dimerization
Source: Natl Sci Rev. 2023 Apr 20;10(9):nwad105. doi: 10.1093/nsr/nwad105 (PMC10575609; doi:10.1093/nsr/nwad105)
Supplement: nwad105_Supplemental_File [file nwad105_supplemental_file.pdf]

Supplementary Information for: Molecular understanding of cation effects on double layer and its significance to CO-CO dimerization

Jia-Bo Le,[†,§] Ao Chen,[‡,§] Yongbo Kuang,[†] and Jun Cheng[*,‡,¶]

†*Ningbo Institute of Materials Technology and Engineering, Chinese Academy of Sciences, Ningbo 315201, China*

‡*State Key Laboratory of Physical Chemistry of Solid Surfaces, iChEM, College of Chemistry and Chemical Engineering, Xiamen University, Xiamen 361005, China*

¶*Innovation Laboratory for Sciences and Technologies of Energy Materials of Fujian Province (IKKEM), Xiamen, China*

§*These authors contribute equally to this work.*

E-mail: chengjun@xmu.edu.cn

Section S1. Modeling of electrified Pt(111)-CO_{ad}/water interfaces

Pt(111) surface was modeled by a $p(4 \times 4)$ periodic slab with 4 atomic layers. Based on the images observed from electrochemical scanning tunneling microscopy (EC-STM),[1] the surface coverage of adsorbed CO on Pt(111) was set to 3/4 ML, with 1/4 ML on top sites and 1/2 ML on hollow sites, forming a (2x2)-3CO adlayer. Both of the two Pt(111) surfaces were adsorbed with CO, and thus there is no net dipole. Large vacuum region was modeled on top of the Pt(111) surface to avoid the interaction of Pt(111) from its images. The overall size of the surface model was $11.246 \times 11.246 \times 40$ Å^3.

Neutral Pt(111)-CO_{ad}/water interface was modeled by fully filling the vacuum region in the Pt(111)-CO_{ad} surface model with water, as shown in Figure S1(a). The density of water in the bulk region was controlled to be $\sim$1 g/cm^3. Note that the model of Figure S1(a) corresponds to the interface structure at the potential of zero charge (PZC) in the context of electrochemistry.[2]

Electric double layers (EDLs) were modeled by introducing cations at the vicinity of the Pt(111)-CO_{ad} surface. In this work, 7 kinds of cations (Li^+, Na^+, Rb^+, Cs^+, Me_4N^+, Et_4N^+ and Pr_4N^+) were employed for modeling EDLs at same surface charge density (σ), as shown in Figure S1(b)-S1(h). The σ of EDLs are controlled by the number of cations at the vicinity of the surfaces, and are set to σ = -14.6 μC/cm^2, i.e. one cation for each surface. Moreover, to study the effect of interfacial ionic concentration on double layer capacitance, two extra Cs-Cl ion pairs were added to the interface model Cs^+(1), as seen in Figure S1(i). It should be mentioned that the Gouy-Chapman layers were not included in modeled interfaces, and thus these EDL models correspond to the high concentration limits in experiment, where surface charges were effectively screened within Helmholtz layers.

Ab initio molecular dynamics (AIMD) simulations for Pt(111)-CO_{ad}/water interfaces were performed by freely available software CP2K.[3] During the computation, 1s electron of

H, 3s, 3p electrons of Cl, 1s, 2s electrons of Li, 2s, 2p electrons of O, 2s, 2p electrons of C, 2s, 2p electrons of N, 2s, 2p, 3s electrons of Na, 4s, 4p, 5s electrons of Rb, 5s, 5p, 6s electrons of Cs and 5d, 6s electrons of Pt were treated as valence, and the rest core electrons were represented by Goedecker-Teter-Hutter (GTH) pseudopotentials.[4,5] The Gaussian basis set was double-ζ with one set of polarisation functions (DZVP),[6] and the energy cutoff was set to 400 Ry.

It is well-known that PBE functional has an incorrect prediction for the most stable CO binding site on the Pt(111) surface. It predicts that CO binds stronger at the hollow site than the top site, which is inconsistent with experiment. In contrast, BLYP functional can correctly predict that the top site of Pt(111) is the most stable adsorption site for CO.[7] Based on those phenomena, BLYP functional was chosen[8–10] to describe the exchange-correlation effects in this work, rather than the PBE functional[11] used in our previous work.[12–14] The dispersion energy was corrected with the Grimmer's D3 method.[15]

The second generation Car-Parrinello molecular dynamics (SGCPMD)[7,16] method was used to sample the structures of interface models. The correction step was obtained by 5 iterations of the orbital transformation (OT) optimization,[17] and the integration time for each step was 0.5 fs. The target temperature was set to 330 K. The Langevin friction coefficient (γ_L) was set to 0.001 fs^{-1}, and the intrinsic friction coefficients (γ_D) were 2.2×10^{-4} fs^{-1} for H_2O, CO and ions. Pt slab was fixed during MD simulations. Due to the large size of these interfaces, only the Γ point in the reciprocal space was used in AIMD simulations. For each AIMD simulation, at least 5 ps (10000 steps) of the trajectory was used to pre-equilibrate the system, and then followed by a production period of 10$\sim$40 ps.

Section S2. Determination of electrode potentials for modeled Pt(111)-CO_{ad}/water interfaces

Electrode potentials (U) of all modeled Pt(111)-CO_{ad}/water interfaces were calculated with the computational standard hydrogen electrode (cSHE) method.[12] The mathematical formulation is

$$e_0 U = -E_{\mathrm{F}}^{(i)} - e_0\phi_{\mathrm{wat}}^{(i)} + \Delta_{\mathrm{dp}}A_{\mathrm{H_3O^+}}^{(w)} - \mu_{\mathrm{H^+}}^{g,\circ} - \Delta E_{\mathrm{zp}}. \qquad (1)$$

In Eqn (1), $E_{\mathrm{F}}^{(i)}$ and $\phi_{\mathrm{wat}}^{(i)}$ denote for the Fermi level of the interface and the electrostatic potential of the bulk water at the interface, respectively. $\Delta_{\mathrm{dp}}A_{\mathrm{H_3O^+}}^{(w)}$ is the deprotonation free energy of $H_3O^+(aq)$ calculated in a pure water model, which has been reported in the previous work,[18] and the recommended value is 15.35 eV. $\mu_{\mathrm{H^+}}^{g,\circ}$ is the standard chemical potential of gas phase proton, and ΔE_{zp} is a correction term for the zero-point energy of an O-H bond in $H_3O^+(aq)$ ion. These two terms are known constants, 15.81 eV and 0.35 eV. Note that electrode potentials calculated with the cSHE method are directly on the scale of SHE.

$E_{\mathrm{F}}^{(i)}$ and $\phi_{\mathrm{wat}}^{(i)}$ in Eqn (1) were obtained by averaging over the AIMD trajectories, and one of every 100 MD configurations was picked for computing the electrode potential. It is shown in Figure S2 both $E_{\mathrm{F}}^{(i)}$ and $-e_0\phi_{\mathrm{wat}}^{(i)}$ are well converged within an uncertainty of 0.1 eV after each AIMD run. The error bars of the computed potentials were estimated by dividing each AIMD trajectory into five evenly-spaced blocks, and then calculating the standard error (SE) of the electrode potentials computed for the five blocks. SE is expressed as SE $= \beta/\sqrt{n}$, where β is the standard deviation and n is the number of samples.

Section S3. Decomposition of Helmholtz capacitance of Pt(111)-CO_{ad}/water interface

It has been shown by Sundararaman and co-workers[19] that the surface adsorbed CO molecules play as a dielectric layer, and thus the Helmholtz capacitance of the Pt(111)-CO_{ad}/water interface can be separated into two components, CO adlayer induced capacitance ($C_{\rm ad}$) and water dielectric screening induced capacitance ($C_{\rm sol}$). The interface potential profile is shown in Figure S3(a). The potential change at interface can be expressed as

$$-\psi_{\rm M} = E_{\rm ad} \cdot l_{\rm ad} + E_{\rm sol} \cdot (l_{\rm H} - l_{\rm ad}) + const., \tag{2}$$

where $E_{\rm ad}$ is the electric field inside the CO adlayer, and $E_{\rm sol}$ represents the electric field generated by the surface charge. $l_{\rm ad}$ and $l_{\rm H}$ are the width of the CO adlayer and Helmholtz layer, respectively. Note that as shown in Figure S3(a), the potential of the bulk solution is set to zero following the convention. The term *const.* is a constant.

$E_{\rm sol}$ in Eqn (2) directly relates to the surface charge density (σ) and the dielectric constant of solvent at interface ($\varepsilon_{\rm sol}$). It can be written as

$$E_{\rm sol} = \frac{\sigma}{\varepsilon_0 \cdot \varepsilon_{\rm sol}}, \tag{3}$$

where ε_0 is the vacuum permittivity. It should be mentioned that in all our modeled interfaces σ is controlled by the net number of cations, i.e. σ = -14.6 μC/cm^2. We find there is no charge transfer occurred between cations and Pt(111)-CO_{ad} surfaces, and it is supported by the following evidence. First, it is shown in Figure S4 there is no density of states observed near the Fermi level for all the cations at interface, indicating no partial charge transfer. chemical bonds formed between cations and metal surfaces. Second, the Mulliken charge analysis (see Table S1) reveals the charges on all the cations are close to +1 e_0 (i.e. $\sim$+0.8 e_0), further confirming that the cations keep their ionic nature at interface. Thirdly,

Figure S3(c) shows that the partition of surface charges on Pt surface (18%) is the same for different cations, indicating the amount of surface charges induced by different cations is the same.

CO is known to play as a dielectric layer, and apparently it separates the surface charge into two parts; charges on Pt surface (σ_{Pt}) and charges on CO adlayer (σ_{CO}), as shown in Figure S3(b), S3(c) and S5. It can be known from Figure S3(c) the partition of surface charge on Pt is 18%. E_{ad} can thus be written as

$$E_{\mathrm{ad}} = \frac{\sigma_{\mathrm{Pt}}}{\varepsilon_0}. \tag{4}$$

Substituting Eqns.(3) and (4) into Eqn. (2), we obtain

$$-\psi_{\mathrm{M}} = \frac{\sigma}{\varepsilon_0 \cdot \varepsilon_{\mathrm{sol}}} \cdot (l_{\mathrm{H}} - l_{\mathrm{ad}}) + \frac{\sigma_{\mathrm{Pt}}}{\varepsilon_0} \cdot l_{\mathrm{ad}} + const.. \tag{5}$$

Taking the derivative of ψ_{M} with respect to σ can give the inverse Helmholtz capacitance ($1/C_{\mathrm{H}}$),

$$\frac{1}{C_{\mathrm{H}}} = \frac{l_{\mathrm{H}} - l_{\mathrm{ad}}}{\varepsilon_0 \cdot \varepsilon_{\mathrm{sol}}} + \frac{l_{\mathrm{ad}} \cdot \sigma_{\mathrm{Pt}}}{\varepsilon_0 \cdot \sigma}. \tag{6}$$

We define the first term in the right hand as the capacitance of usual dielectric response of solvent in the Helmholtz layer (C_{sol}),

$$\frac{1}{C_{\mathrm{sol}}} = \frac{l_{\mathrm{H}} - l_{\mathrm{ad}}}{\varepsilon_0 \cdot \varepsilon_{\mathrm{sol}}}, \tag{7}$$

and define the second term on the right hand of Eqn. (6) as a new capacitance C_{ad} from the CO adlayer,

$$\frac{1}{C_{\mathrm{ad}}} = \frac{l_{\mathrm{ad}} \cdot \sigma_{\mathrm{Pt}}}{\varepsilon_0 \cdot \sigma}. \tag{8}$$

Combining Eqns. (6), (7) and (8), we can write

$$\frac{1}{C_{\mathrm{H}}} = \frac{1}{C_{\mathrm{sol}}} + \frac{1}{C_{\mathrm{ad}}}, \tag{9}$$

and it is known that the Helmholtz layer of Pt(111)-CO_{ad}/water interface can be regarded as two capacitors (C_{ad} and C_{sol}) connected in series.

In view that both of l_{ad} and $\sigma_{\mathrm{pt}}/\sigma$ can be extracted from Figure S3(c) (l_{ad} = 1.48 Å and $\sigma_{\mathrm{pt}}/\sigma$ = 18%), C_{ad} is thus easily obtained, 44.5 μF/cm^2. Note that the formulation of interface potential change does not take into account the work function change of Pt(111)-CO_{ad} surface at different surface charge densities. It is because we find the pattern of CO adlayer has almost no change at different conditions, as shown in Figure S6. Accordingly, it is revealed that the work function of the Pt(111)-CO_{ad} surface extracted from AIMD interface models ($\Phi_{\mathrm{Pt(111)-CO_{ad}}}$) is only changed by ∼0.1 eV (see Figure S7) by decreasing the surface charge density from 0 to -14.6 μC/cm^2. The change in $\Phi_{\mathrm{Pt(111)-CO_{ad}}}$ can be attributed to the change of CO bond length, which is dependent on the surface charge density, while we think this small difference in $\Phi_{\mathrm{Pt(111)-CO_{ad}}}$ will not clearly change the capacitance of the Pt(111)-CO_{ad}/water interface, and thus is omitted. Furthermore, it should be noted that the magnitude of C_{ad} is directly relevant to the surface charge partition on Pt (σ_{Pt}) according to the expression shown in Eqn. (8). As shown in Figure S14, we find σ_{Pt} is dependent on the surface coverage of CO (θ_{CO}), and it indicates the θ_{CO} is an important property for understanding the capacitance of metal-CO_{ad}/water interfaces.

Section S4. Comparison of surface charge distributions on $Pt(111)\text{-}CO_{ad}$ and $Cu(100)\text{-}CO_{ad}$ surfaces

The distributions of surface charges on the $Pt(111)\text{-}CO_{ad}$ and $Cu(100)\text{-}CO_{ad}$ surfaces were compared. Pt(111) and Cu(100) surfaces were modeled by 6×6 periodic slabs with 4 atomic layers. The surface coverage of CO was 1 ML, and all CO molecules were adsorbed on the top sites, as shown in Figure S12(a) and S12(b). The structures of two surfaces were both optimized with DFT calculations at the neutral condition. During structural optimization, the bottom two metal layers were fixed, and to avoid the dipole interaction with periodic images, the periodicity in the surface normal direction of these models was removed. PBE-D3 functional[11,15] and BFGS minimizer were employed for these calculations.

Then, one excess electron was introduced to the surface model, and the excess electron on metal surfaces was compensated by a counter charge plane, which was modeled by an array of classical point charges with the same amount of charge. The distribution of planar average of excess electron density ($\Delta\rho_\mathrm{e}$) shown in Figure S12(c) was calculated with

$$\Delta\rho_\mathrm{e} = \rho_\mathrm{charged} - \rho_\mathrm{neutral}, \tag{10}$$

and the amount of surface charge (q, see Figure S12(d)) was calculated by integrating the charge from the bulk of bulk to vacuum,

$$q(z) = \int_{metal}^{vacuum} \Delta\rho_\mathrm{e} * S dz, \tag{11}$$

where S is surface area.

Section S5. Computation of CO-CO dimerization on Cu(100) surface

CO dimerization reaction was computed on the Cu(100) surface at various conditions (different surface charge densities; with or without coordination of water and Cs^+ ion, as shown in Figure S13). A 6×6 supercell with four atomic layers was used to represent the Cu(100) surface. In the absence of cations, the model size is $15.142 \times 15.142 \times 35$ Å^3. Two different surface charge densities were considered, -3.5 μC/cm^2 and -7 μC/cm^2. The excess charges on the Cu(100) surfaces were compensated by a neutralizing background as implemented in the standard Ewald sum under periodic boundary conditions (PBC). To study the effect of hydrogen bond, each adsorbed CO was bonded with one water molecule as an approximation, and the oxygen atoms of the coordinated water molecules were fixed during the geometry optimization. In the presence of a solvated Cs^+, the slab carries a net dipole and the Martyna-Tuckerman poisson solver was used to remove the spurious dipole-dipole interaction between the slab and its images. At this condition, the z-coordinate of the model was extended from 35 Å to 60 Å to avoid the overlapping of electron density between the slab and its images.

All the static DFT calculations were performed with CP2K. The 3d, 4s electrons of Cu were treated as valence. A matrix diagonalization procedure was used for the wave function optimization and the self-consistent field (SCF) convergence was facilitated by Fermi smearing with the electronic temperature of 300 K. PBE-D3 functional[11,15] was used to describe the exchange-correlation effects in these calculations. Note that no liquid water was included in the model and the reaction was calculated using static structural optimization approach. We are aware that these computational settings neglect important effects in electrochemical environments such as dynamic solvation. However, explicit treatment of electrified interfaces and free energy calculation at the AIMD level are not feasible due to the high computational costs. We hope that comparison of the relative energies at different

conditions is still physically meaningful to help understand the effects of surface charge and coordination of water and ions, even with the much simplified models.

Supplementary figures

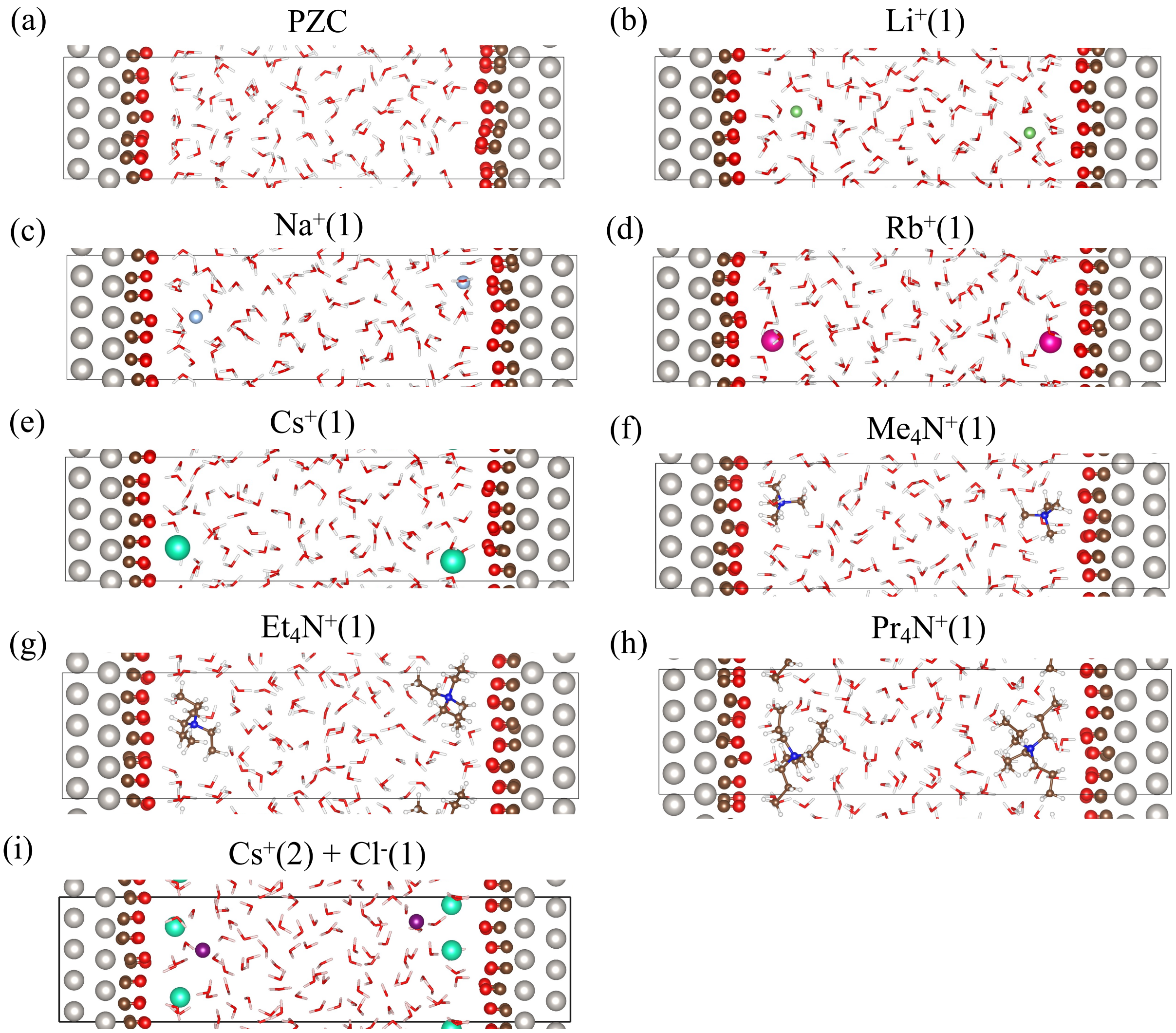

Figure S1: Models of Pt(111)-CO_{ad}/water interfaces. (a) at PZC, (b) with Li^+, (c) with Na^+, (d) with Rb^+, (e) with Cs^+, (f) with Me_4N^+, (g) with Et_4N^+, (h) with Pr_4N^+, and (i) with Cs^+ and extra Cs^+-Cl^- pairs. Pt, C, O, H, N, Li, Na, Rb, Cs, and Cl atoms are colored by grey, brown, red, white, blue, green, lightsteelblue, deeppink, cyan and purple, respectively. The rectangles indicate the periodic boundary conditions of these models.

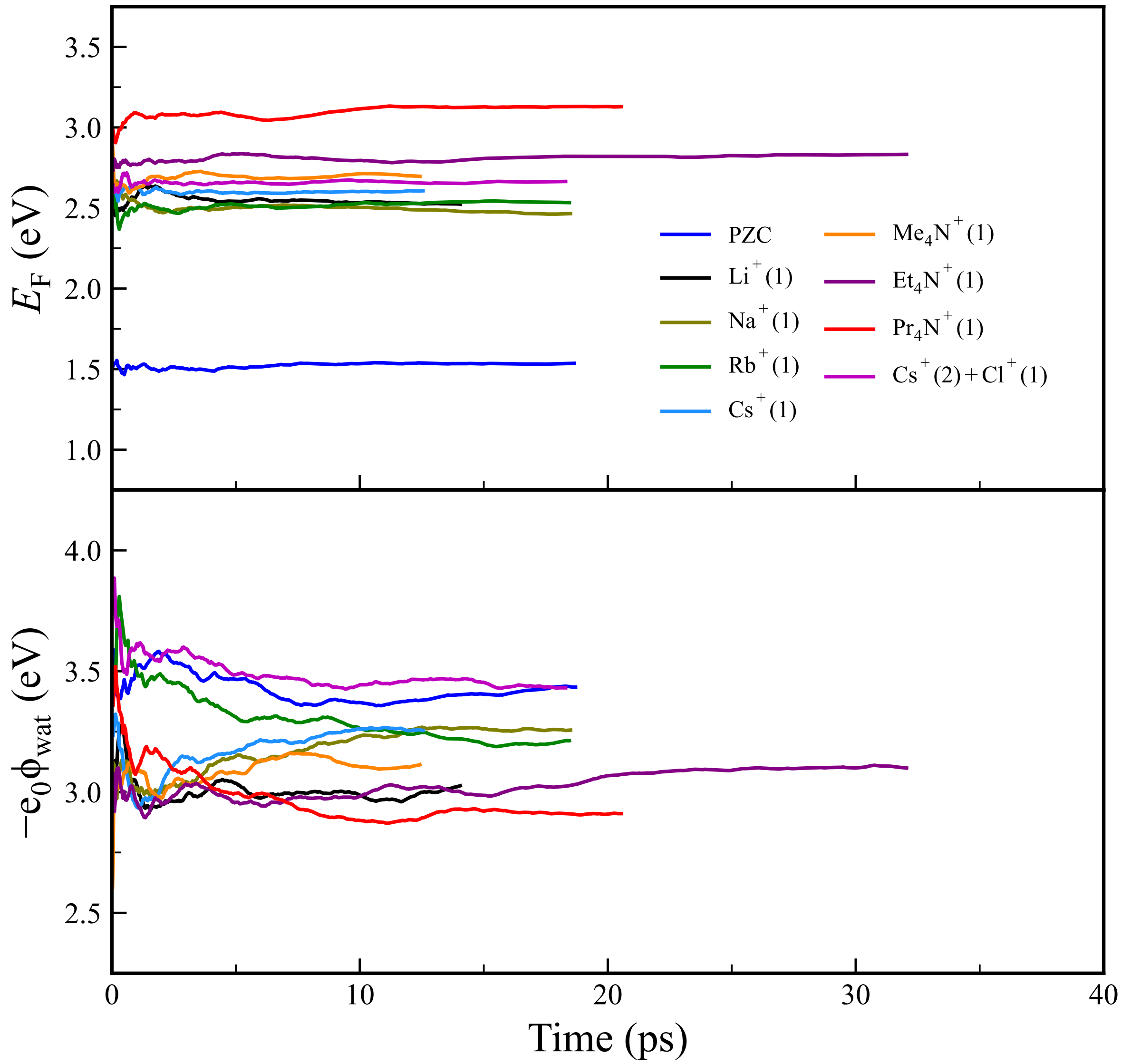

Figure S2: Time accumulative averages of Fermi levels ($E_F^{(i)}$) of interfaces and electrostatic potential energies of bulk water ($-e_0\phi_{\rm wat}^{(i)}$) from different Pt(111)-CO_{ad}/water interfaces.

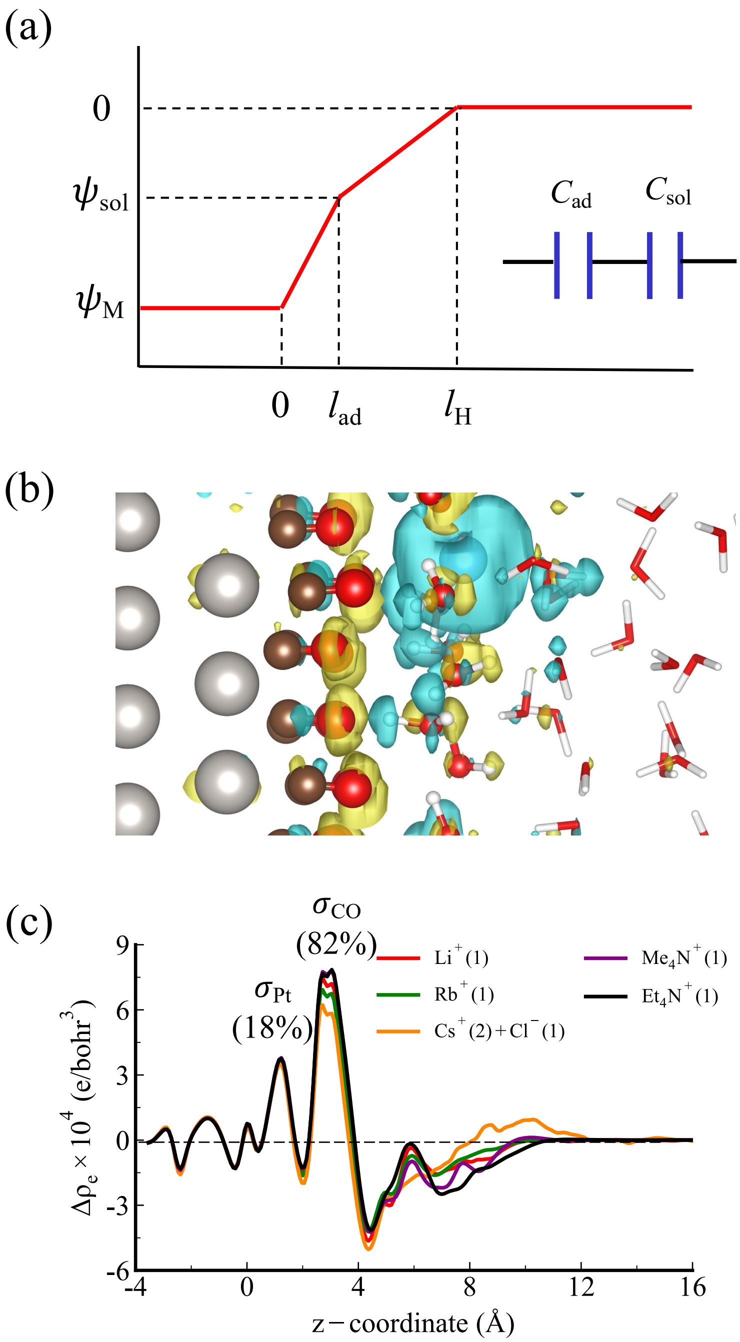

Figure S3: (a) Potential distribution profile at Pt(111)-CO_{ad}/water interface. The Helmholtz capacitance (C_H) is decomposed into two components, CO adlayer induced capacitance (C_{ad}) and water dielectric screening induced capacitance (C_{sol}). (b) The charge excess at the Pt(111)-CO_{ad}/water interface. The iso-surfaces colored in cyan and yellow represent electron depletion and accumulation regions, respectively. (c) Planar averaged charge excess ($\Delta\rho_e$)) distribution at interface. It is calculated with $\Delta\rho_e = \rho_{interface} - \rho_{Pt(111)-CO_{ad}} - \rho_{water} - \rho_{ion}$, where $\rho_{interface}$, $\rho_{Pt(111)-CO_{ad}}$, ρ_{water}, and ρ_{ion} denote for the electronic densities of interface, neutral Pt(111)-CO_{ad} surface, water, and neutral ions, respectively.

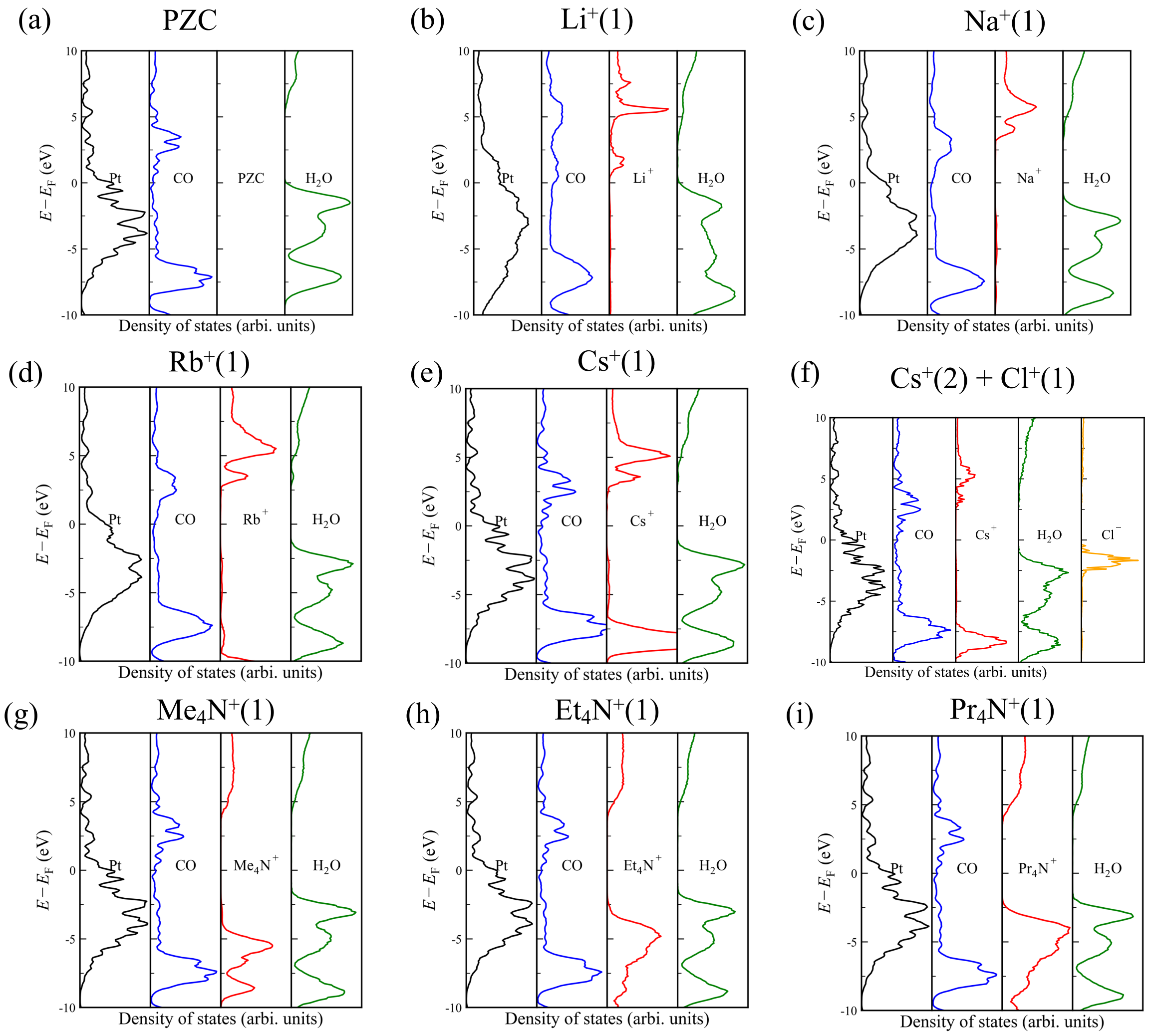

Figure S4: Electronic density of states (DOS) of Pt(111)-CO_{ad}/water interfaces at different conditions. The energy levels of states for each interface are all referenced to their respective Fermi level (E_F).

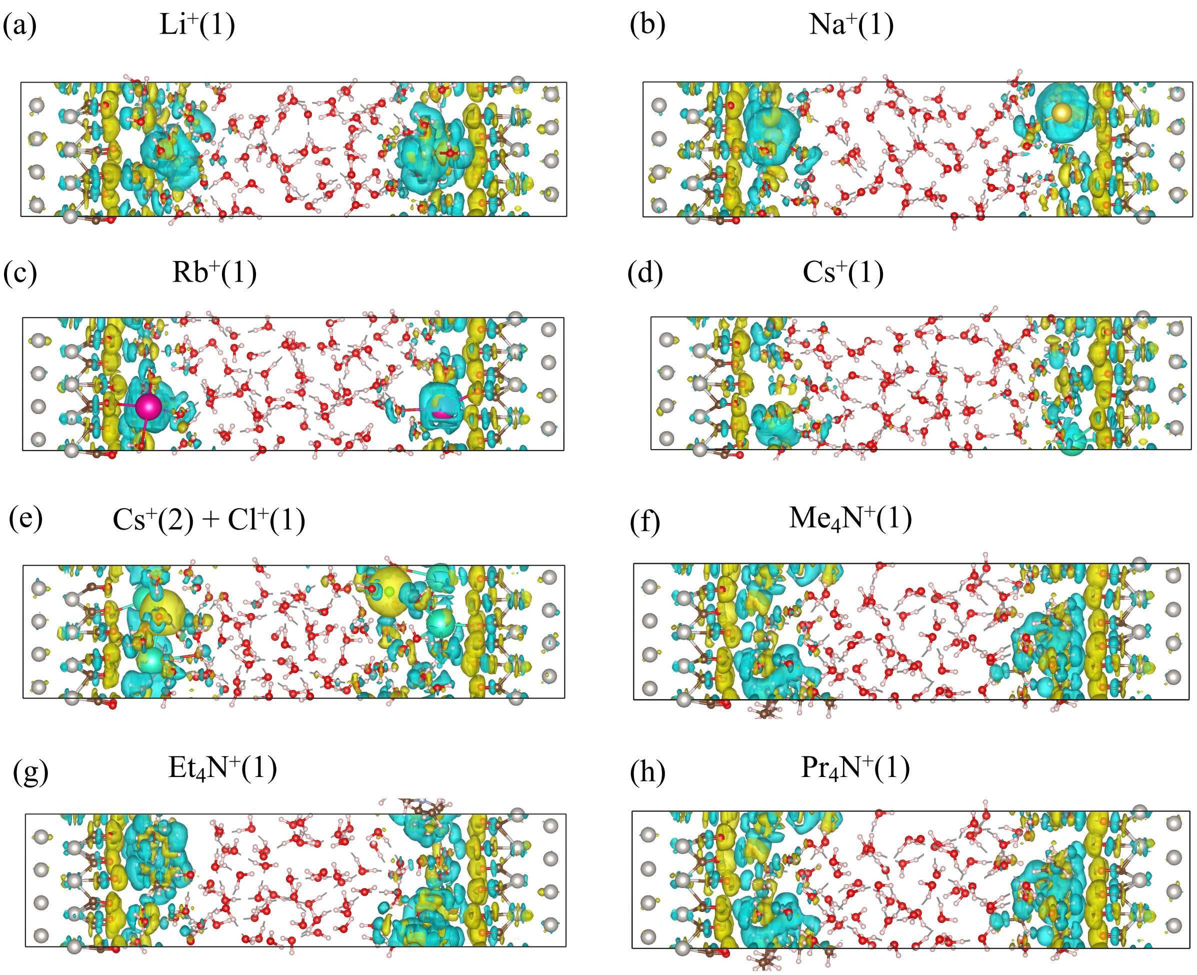

Figure S5: Charge excesses ($\Delta\rho_{\mathrm{e}}$) at electrified Pt(111)-CO_{ad}/water interfaces. $\Delta\rho_{\mathrm{e}}$ is calculated with $\Delta\rho_{\mathrm{e}} = \rho_{\mathrm{interface}} - \rho_{\mathrm{Pt(111)-CO_{ad}}} - \rho_{\mathrm{water}} - \rho_{\mathrm{ion}}$, where $\rho_{\mathrm{interface}}$, $\rho_{\mathrm{Pt(111)-CO_{ad}}}$, ρ_{water}, and ρ_{ion} denote for the electronic densities of the interface, neutral Pt(111)-CO_{ad} surface, water, and neutral ions, respectively. The iso-surfaces colored in cyan and yellow represent electron depletion and accumulation regions, respectively.

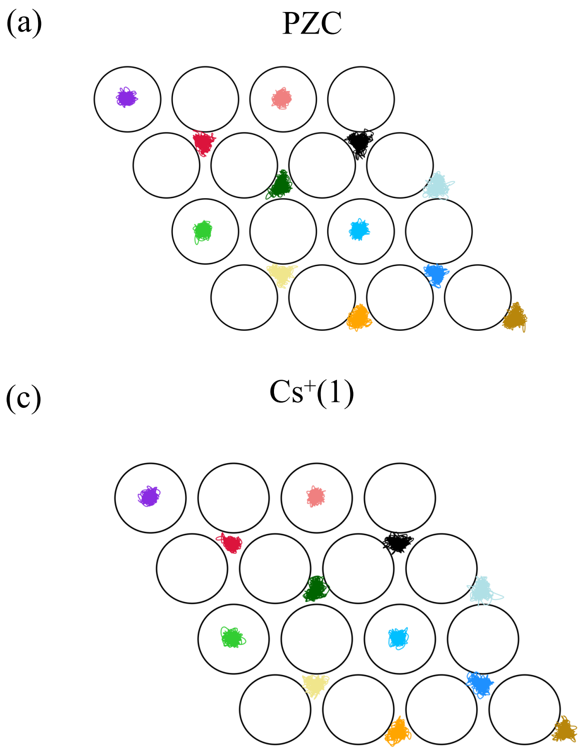

Figure S6: Trajectories of adsorbed CO on Pt(111) surfaces at different conditions during AIMD simulations. Each CO is distinguished by a different color.

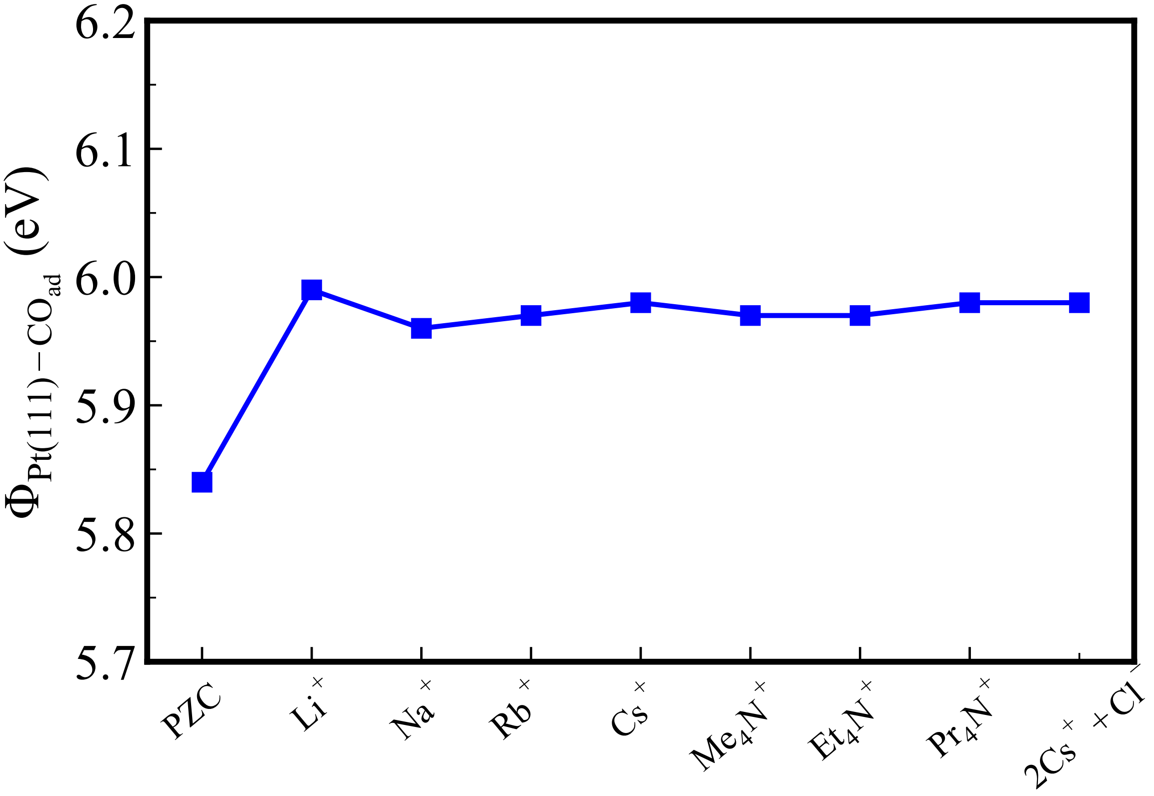

Figure S7: Work functions of Pt(111)-CO_{ad} surfaces ($\Phi_{Pt(111)-CO_{ad}}$) at different conditions. The surface structures used for work function calculations are extracted from AIMD trajectories.

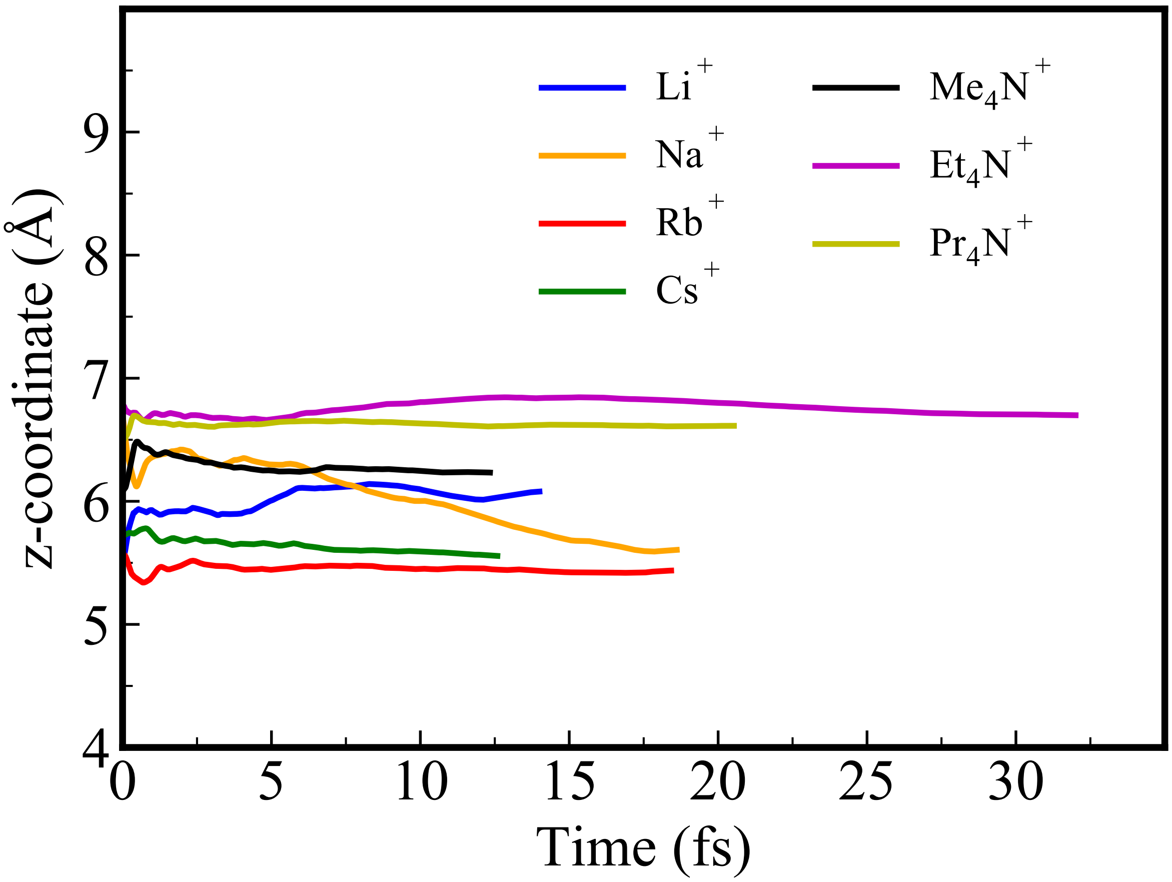

Figure S8: Time accumulative averages of ion positions at Pt(111)-CO_{ad}/water interfaces. The results are averaged by two symmetrical interfaces. The z-coordinates of ions have been referenced to the position of the top Pt layer. The coordinates of N atoms are used to represent the positions of alkyl-ammonium ions.

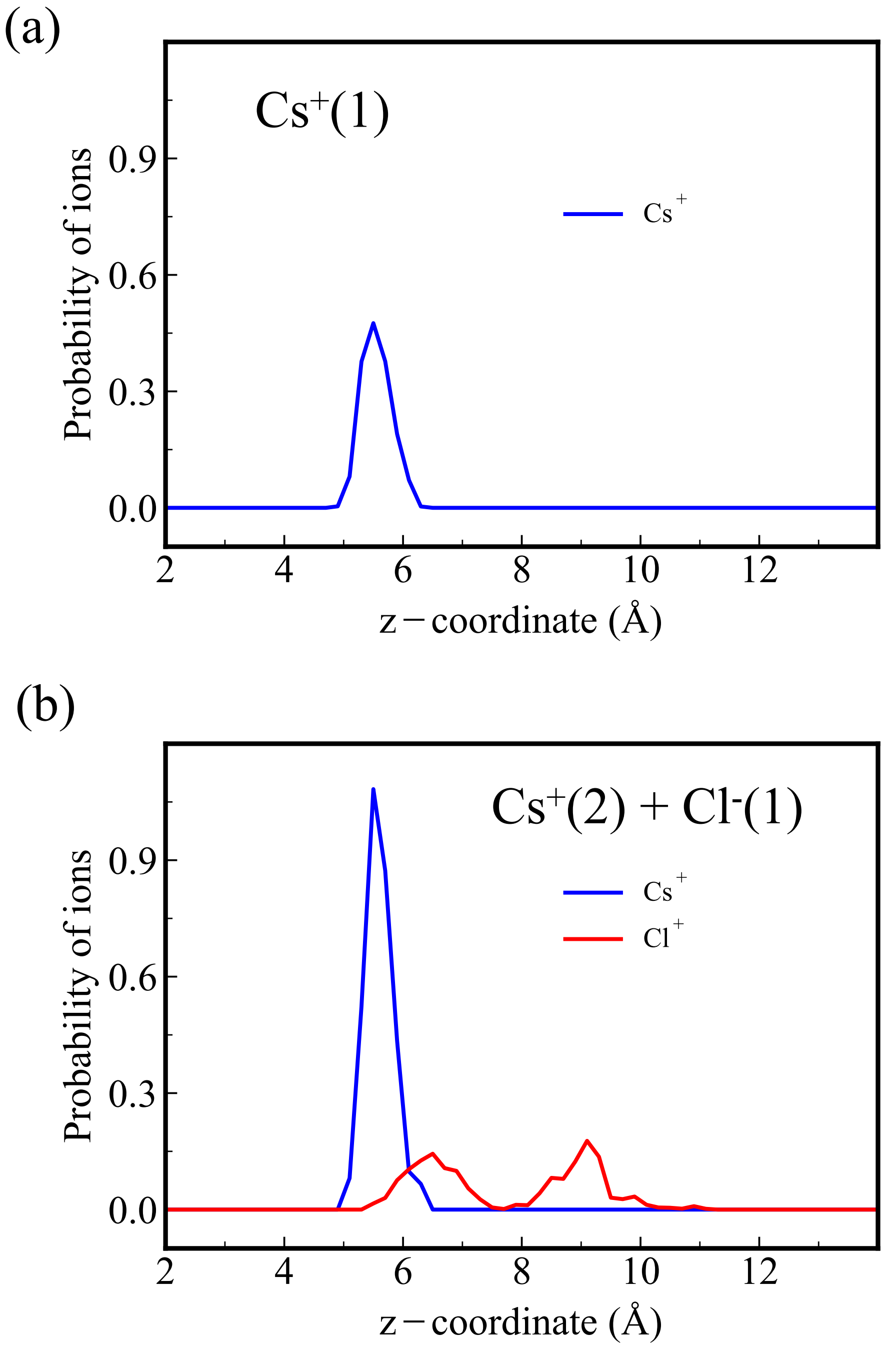

Figure S9: (a) The probability distribution of Cs^+ ions at interface model of $Cs^+(1)$. (b) The probability distributions of Cs^+ and Cl^- ions at interface model of $Cs^+(2)$ + Cl^-.

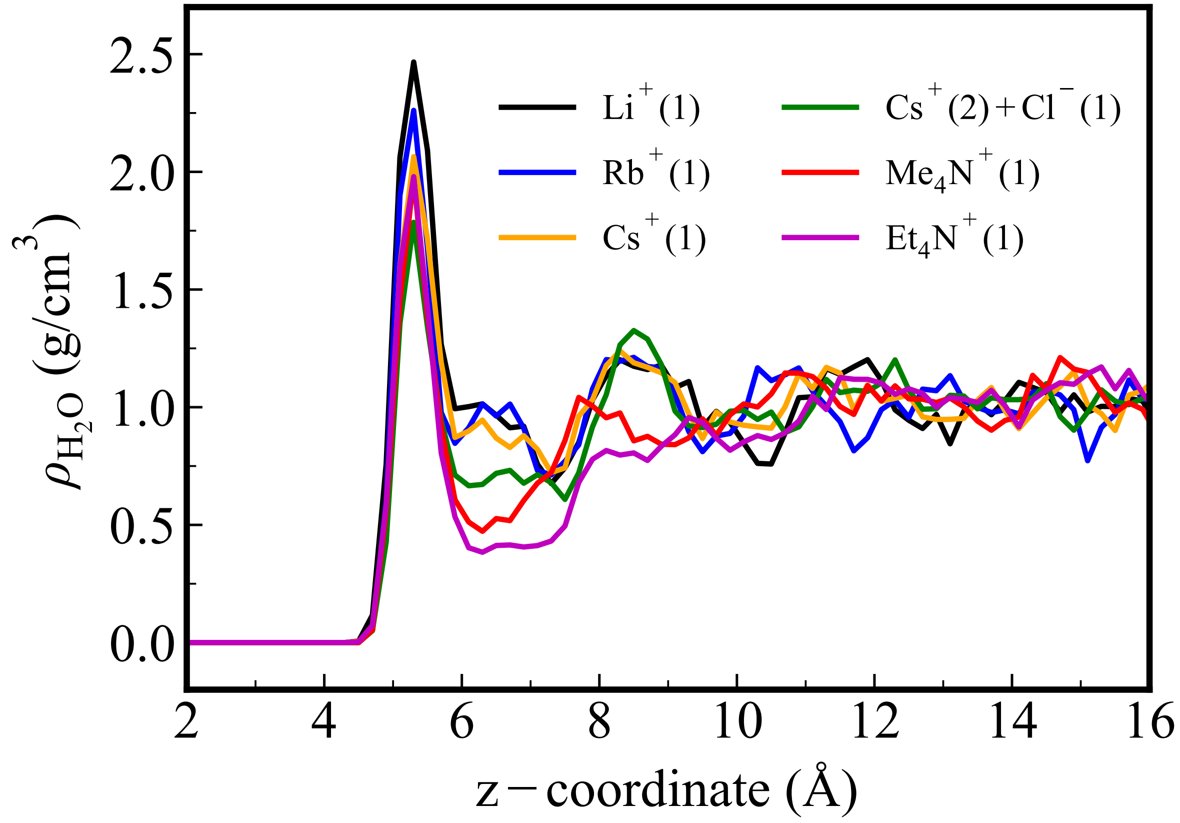

Figure S10: Density distributions of water (ρ_{H_2O}) along the surface normal.

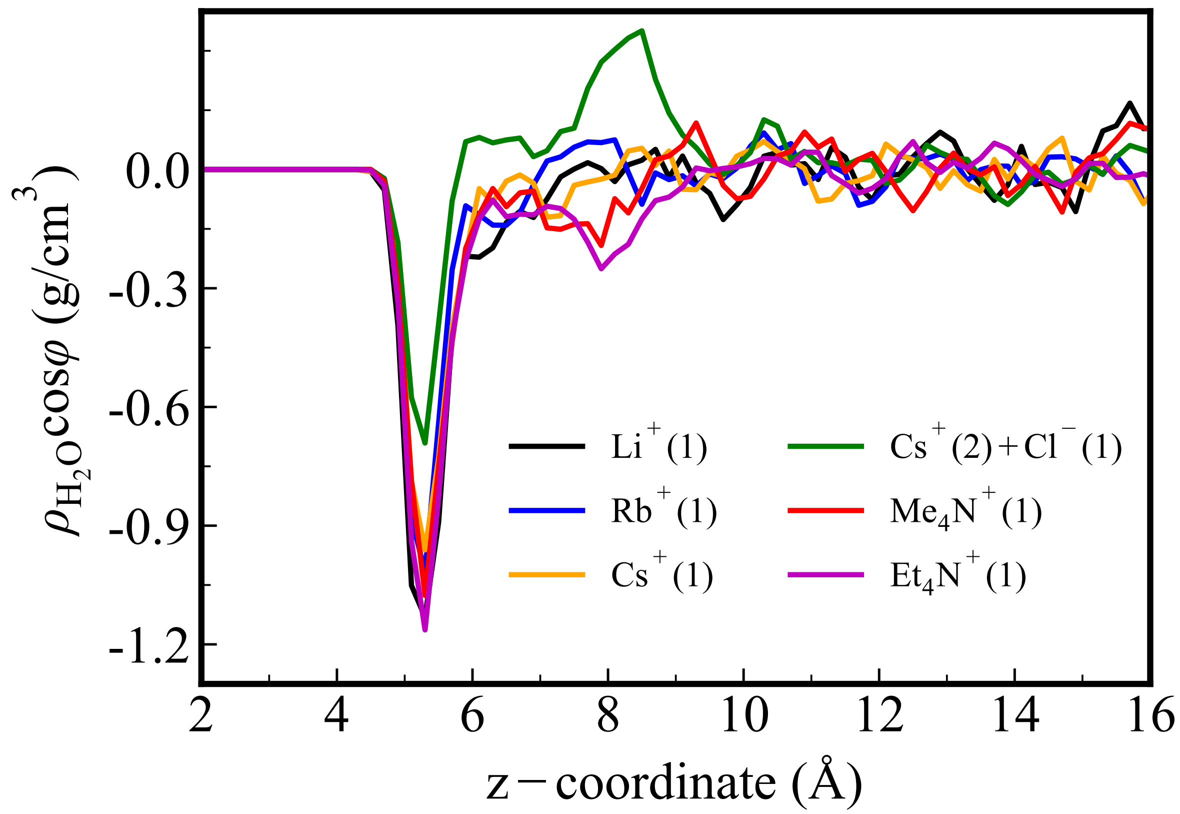

Figure S11: Distribution profiles of dipole orientation of water ($\rho_{H_2O}cos(\varphi)$) along the surface normal. φ is defined as the angle between the bisector of water and the surface normal.

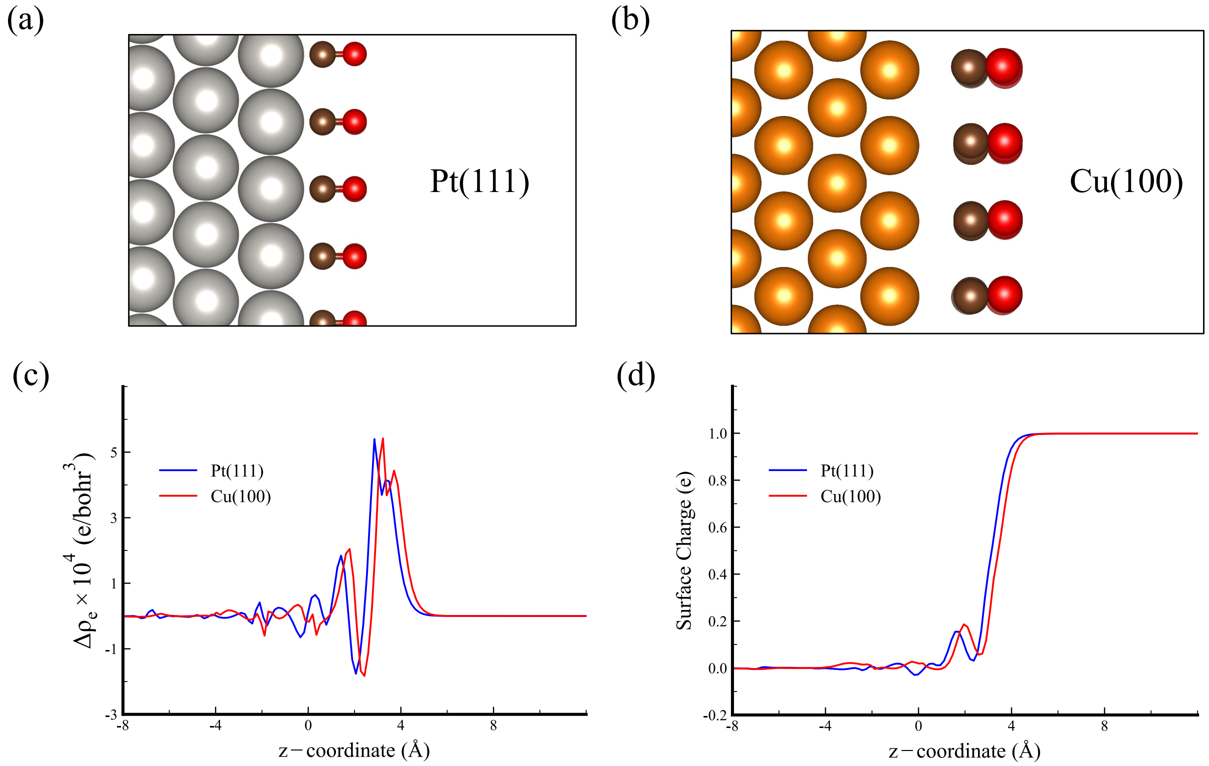

Figure S12: (a) Atomic model of a CO-adsorbed Pt(111) surface. (b) Atomic model of a CO-adsorbed Cu(100) surface. (c) Distributions of excess electrons at Pt(111)-CO_{ad} and Cu(100)-CO_{ad} surfaces. (d) Distributions of integrated surface charge amount at Pt(111)-CO_{ad} and Cu(100)-CO_{ad} surfaces.

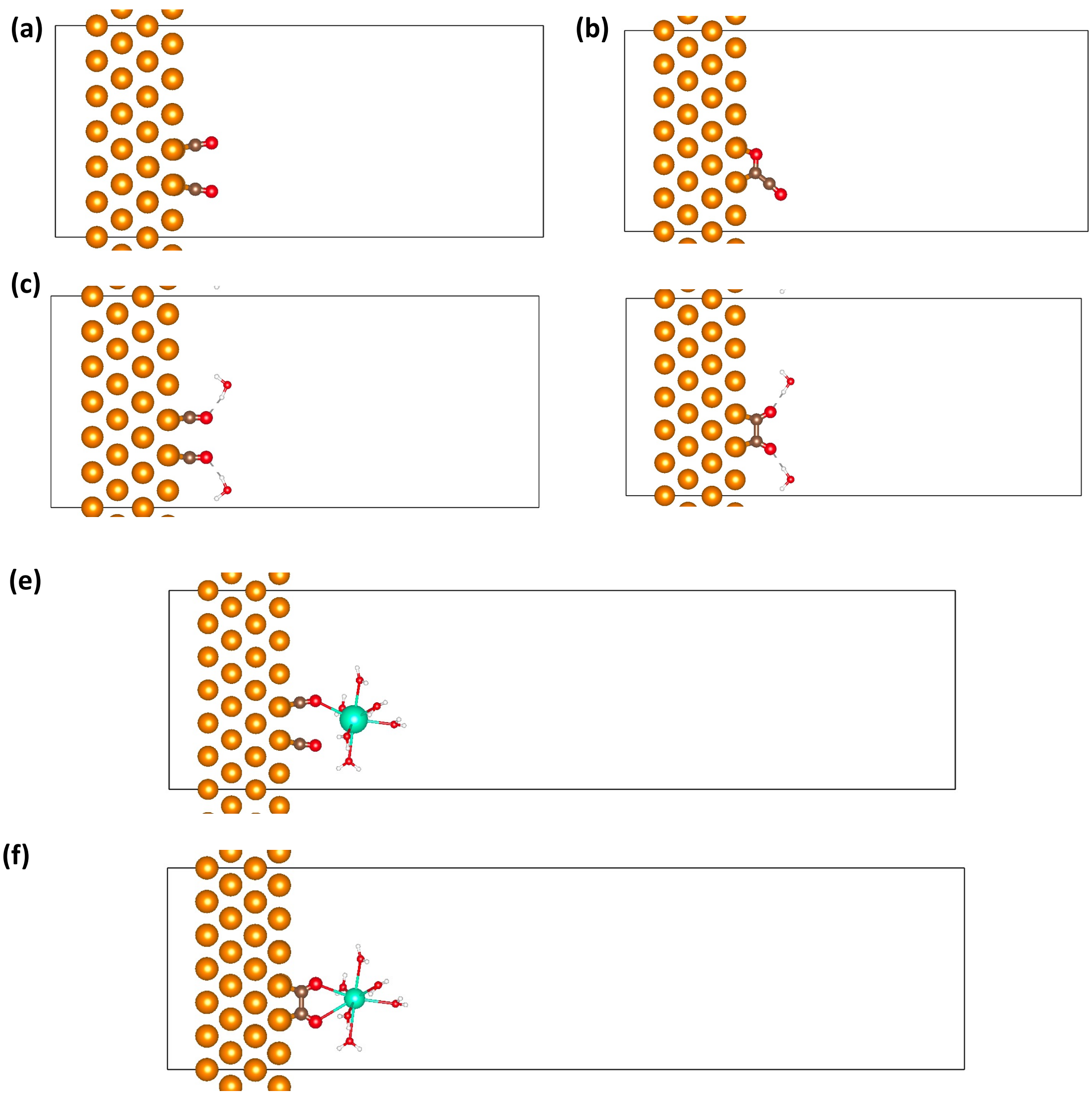

Figure S13: Models of CO* and OCCO* adsorbed on Cu(100) surfaces at different conditions.

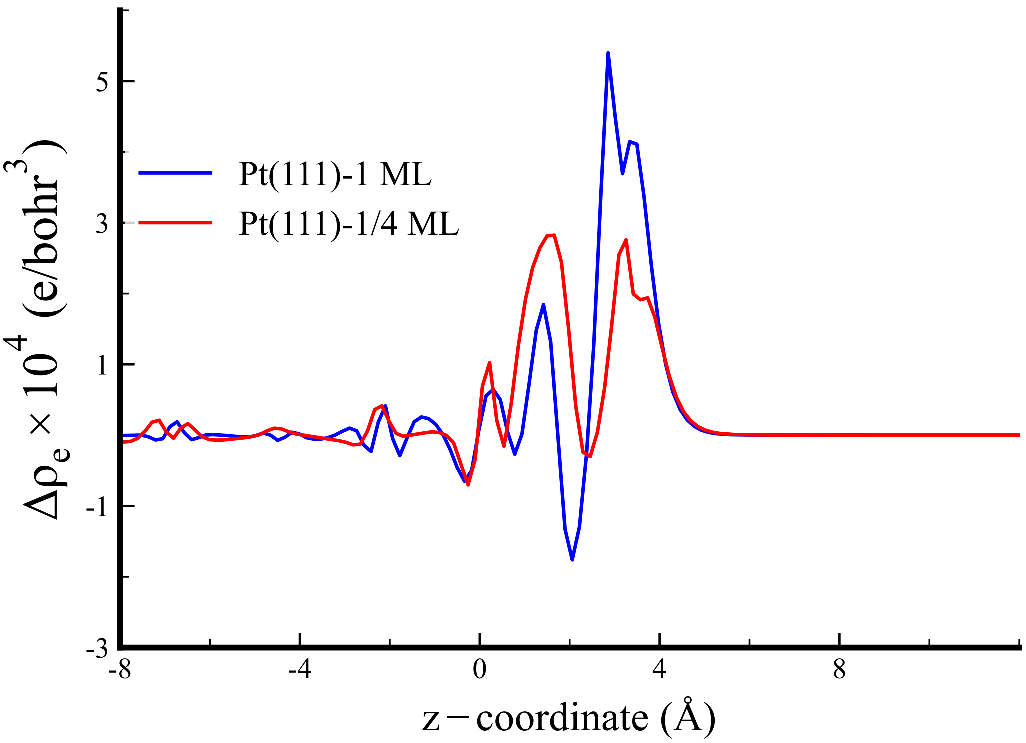

Figure S14: Comparison of distributions of excess electrons at Pt(111)-CO_{ad} surfaces by varying the surface coverage of CO.

Supplementary table

Table S1: Calculated Mulliken charges of ions at Pt(111)-CO_{ad}/water interfaces. All the charges were obtained by averaging over 5 representative interface configurations.

Ions at interfaces	Mulliken charges (e_0)
Li^+	+0.71
Na^+	+0.83
Rb^+	+0.78
Cs^+	+0.89
Me_4N^+	+0.84
Et_4N^+	+0.83
Pr_4N^+	+0.83

References

(1) Villegas, I.; Weaver, M. J. Carbon monoxide adlayer structures on platinum (111) electrodes: A synergy between in-situ scanning tunneling microscopy and infrared spectroscopy. *J. Chem. Phys.* **1994**, *101*, 1648–1660.

(2) Boronat-González, A.; Herrero, E.; Feliu, J. M. Determination of the potential of zero charge of Pt/CO electrodes using an impinging jet system. *J. Solid State Electrochem.* **2020**, *24*, 2871–2881.

(3) VandeVondele, J.; Krack, M.; Mohamed, F.; Parrinello, M.; Chassaing, T.; Hutter, J. Quickstep: Fast and accurate density functional calculations using a mixed Gaussian and plane waves approach. *Comput. Phys. Commun.* **2005**, *167*, 103.

(4) Goedecker, S.; Teter, M.; Hutter, J. Separable dual-space Gaussian pseudopotentials. *Phys. Rev. B* **1996**, *54*, 1703.

(5) Hartwigsen, C.; Goedecker, S.; Hutter, J. Relativistic separable dual-space Gaussian pseudopotentials from H to Rn. *Phys. Rev. B* **1998**, *58*, 3641.

(6) VandeVondele, J.; Hutter, J. Gaussian basis sets for accurate calculations on molecular systems in gas and condensed phases. *J. Chem. Phys.* **2007**, *127*, 114105.

(7) Lan, J.; Hutter, J.; Iannuzzi, M. First-Principles Simulations of an Aqueous CO/Pt(111) Interface. *J. Phys. Chem. C* **2018**, *122*, 24068.

(8) Lee, C.; Yang, W.; Parr, R. G. Development of the Colle-Salvetti correlation-energy formula into a functional of the electron density. *Phys. Rev. B* **1988**, *37*, 785.

(9) Becke, A. D. Density-functional thermochemistry. I. The effect of the exchange-only gradient correction. *J. Chem. Phys.* **1998**, *96*, 2155.

(10) Alaei, M.; Akbarzadeh, H.; Gholizadeh, H.; De Gironcoli, S. CO/Pt (111): GGA density functional study of site preference for adsorption. *Phys. Rev. B* **2008**, *77*, 085414.

(11) Perdew, J. P.; Burke, K.; Ernzerhof, M. Generalized Gradient Approximation Made Simple. *Phys. Rev. Lett.* **1996**, *77*, 3865.

(12) Le, J.; Iannuzzi, M.; Cuesta, A.; Cheng, J. Determining potentials of zero charge of metal electrodes versus the standard hydrogen electrode from based on density-functional-theory-based molecular dynamics. *Phys. Rev. Lett.* **2017**, *119*, 16801.

(13) Le, J.-B.; Fan, Q.-Y.; Li, J.-Q.; Cheng, J. Molecular origin of negative component of Helmholtz capacitance at electrified Pt(111)/water interface. *Sci. Adv.* **2020**, *6*, eabb1219.

(14) Le, J.-B.; Chen, A.; Li, L.; Xiong, J.-F.; Lan, J.; Liu, Y.-P.; Iannuzzi, M.; Cheng, J. Modeling Electrified Pt(111)-Had /Water Interfaces from Ab Initio Molecular Dynamics. *JACS Au* **2021**, *1*, 569–577.

(15) Grimme, S.; Antony, J.; Ehrlich, S.; Krieg, H. A consistent and accurate ab initio parametrization of density functional dispersion correction (DFT-D) for the 94 elements H-Pu. *J. Chem. Phys.* **2010**, *132*, 154104.

(16) Kühne, T. D.; Krack, M.; Mohamed, F. R.; Parrinello, M. Efficient and accurate car-parrinello-like approach to born-oppenheimer molecular dynamics. *Phys. Rev. Lett.* **2007**, *98*, 66401.

(17) VandeVondele, J.; Hutter, J. An efficient orbital transformation method for electronic structure calculations. *J. Chem. Phys.* **2003**, *118*, 4365–4369.

(18) Cheng, J.; Liu, X.; VandeVondele, J.; Sulpizi, M.; Sprik, M. Redox potentials and acidity constants from density functional theory based molecular dynamics. *Acc. Chem. Res.* **2014**, *47*, 3522.

(19) Sundararaman, R.; Figueiredo, M. C.; Koper, M. T. M.; Schwarz, K. A. Electrochemical

Capacitance of CO-Terminated Pt(111) Dominated by the CO–Solvent Gap. *J. Phys. Chem. Lett.* **2017**, *8*, 5344.
